# The Origins of Ashkenaz, Ashkenazic Jews, and Yiddish

**DOI:** 10.3389/fgene.2017.00087

**Published:** 2017-06-21

**Authors:** Ranajit Das, Paul Wexler, Mehdi Pirooznia, Eran Elhaik

**Affiliations:** ^1^Manipal Centre for Natural Sciences, Manipal UniversityManipal, India; ^2^Department of Linguistics, Tel Aviv UniversityTel-Aviv, Israel; ^3^Department of Psychiatry and Behavioral Sciences, Johns Hopkins UniversityBaltimore, MD, United States; ^4^Department of Animal and Plant Sciences, University of SheffieldSheffield, United Kingdom

**Keywords:** Yiddish, Ashkenazic Jews, Ashkenaz, geographic population structure (GPS), Archaeogenetics, Rhineland hypothesis, ancient DNA

## Abstract

Recently, the geographical origins of Ashkenazic Jews (AJs) and their native language Yiddish were investigated by applying the Geographic Population Structure (GPS) to a cohort of exclusively Yiddish-speaking and multilingual AJs. GPS localized most AJs along major ancient trade routes in northeastern Turkey adjacent to primeval villages with names that resemble the word “Ashkenaz.” These findings were compatible with the hypothesis of an Irano-Turko-Slavic origin for AJs and a Slavic origin for Yiddish and at odds with the Rhineland hypothesis advocating a Levantine origin for AJs and German origins for Yiddish. We discuss how these findings advance three ongoing debates concerning (1) the historical meaning of the term “Ashkenaz;” (2) the genetic structure of AJs and their geographical origins as inferred from multiple studies employing both modern and ancient DNA and original ancient DNA analyses; and (3) the development of Yiddish. We provide additional validation to the non-Levantine origin of AJs using ancient DNA from the Near East and the Levant. Due to the rising popularity of geo-localization tools to address questions of origin, we briefly discuss the advantages and limitations of popular tools with focus on the GPS approach. Our results reinforce the non-Levantine origins of AJs.

## Background

The geographical origin of the Biblical “Ashkenaz,” Ashkenazic Jews (AJs), and Yiddish, are among the longest standing questions in history, genetics, and linguistics.

Uncertainties concerning the meaning of “Ashkenaz” arose in the Eleventh century when the term shifted from a designation of the Iranian Scythians to become that of Slavs and Germans and finally of “German” (Ashkenazic) Jews in the Eleventh to Thirteenth centuries (Wexler, 1993). The first known discussion of the origin of German Jews and Yiddish surfaced in the writings of the Hebrew grammarian Elia Baxur in the first half of the Sixteenth century (Wexler, 1993).

It is well established that history is also reflected in the DNA through relationships between genetics, geography, and language (e.g., Cavalli-Sforza, 1997; Weinreich, 2008). Max Weinreich, the doyen of the field of modern Yiddish linguistics, has already emphasized the truism that the history of Yiddish mirrors the history of its speakers. These relationships prompted Das et al. (2016) to address the question of Yiddish origin by analyzing the genomes of Yiddish-speaking AJs, multilingual AJs, and Sephardic Jews using the Geographical Population Structure (GPS), which localizes genomes to where they experienced the last major admixture event. GPS traced nearly all AJs to major ancient trade routes in northeastern Turkey adjacent to four primeval villages whose names resemble “Ashkenaz:” İşkenaz (or Eşkenaz), Eşkenez (or Eşkens), Aşhanas, and Aschuz. Evaluated in light of the Rhineland and Irano-Turko-Slavic hypotheses (Das et al., 2016, Table 1) the findings supported the latter, implying that Yiddish was created by Slavo-Iranian Jewish merchants plying the Silk Roads. We discuss these findings from historical, genetic, and linguistic perspectives and calculate the genetic similarity of AJs and Middle Eastern populations to ancient genomes from Anatolia, Iran, and the Levant. We lastly review briefly the advantages and limitation of bio-localization tools and their application in genetic research.

**Table 1:** Major open questions regarding the origin of the term “Ashkenaz,” AJs, and Yiddish as explained by two competing hypotheses.

**Open questions**	**Rhineland hypothesis**	**Irano-Turko-Slavic hypothesis**	**Evidence in favor of the Irano-Turko-Slavic hypothesis**
The term “Ashkenaz”	Originally affiliated with the people living north of Biblical Israel (Aptroot, 2016) or north of the Black Sea (Wexler, 1991). Used in Hebrew and Yiddish sources from the Eleventh century onward to denote a region in what is now roughly Southern Germany (Wexler, 1991; Aptroot, 2016).	Denotes an Iranian people “near Armenia,” presumably Scythians known as *aškuza, ašguza*, or *išguza* in Assyrian inscriptions of the early Seventh century B.C. (Wexler, 2012, 2016).	GPS analysis uncovered four primeval villages in northeastern Turkey whose names resemble “Ashkenaz,” at least one of which predates any major Jewish settlement in Germany (Das et al., 2016). “Ashkenaz” is thereby a placename associated with the Near East and its inhabitants both Jews and non-Jews.
The ancestral origin of Ashkenazic Jews	Judaean living in Judaea until 70 A.D. who were exiled by the Romans (King, 2001) and remained in relative isolation from neighboring non-Jewish communities during and after the Diaspora (Hammer et al., 2000; Ostrer, 2001). This scenario has no historical (Sand, 2009) nor genetic support (Figure 1B) (e.g., Elhaik, 2013, 2016; Xue et al., 2017).	A minority of Judaean emigrants and a majority of Irano-Turko-Slavic converts to Judaism (Wexler, 2012).	AJs exhibit high genetic similarity to populations living in Turkey and the Caucasus (Das et al., 2016). All bio-location analyses predicted AJs to Turkey (Figure 1A). Ancient DNA analyses provide strong evidence of the Iranian Neolithic ancestry of AJs (Figure 1B) (Lazaridis et al., 2016).
The arrival of Jews to German lands	After the arrival of Palestinian Jews to Roman lands, Jewish merchants and soldiers arrived to German lands with the Roman army and settled there (King, 2001). This scenario has no historical support (Wexler, 1993; Sand, 2009).	Jews from the Khazar Empire and the former Iranian Empire plying the old Roman trade routes (Rabinowitz, 1945, 1948) and Silk Roads began to settle in the mixed Germano-Sorbian lands during the first Millennium (Sand, 2009; Wexler, 2011).	Ashkenazic Jews were predicted to a Near Eastern hub of ancient trade routes that connected Europe, Asia, and the northern Caucasus (Das et al., 2016). The findings imply that migration to Europe took place initially through trade routes going west and later through Khazar lands.
Yiddish's emergence in the 9th century	Between the Ninth and Tenth centuries, French- and Italian-speaking Jewish immigrants adopted and adapted the local German dialects (Weinreich, 2008).	Upon arrival to German lands, Western and Eastern Slavic went through a relexification to German, creating what became known as Yiddish (Wexler, 2012).	Xue et al.'s (2017) inferred “admixture time” of 960–1,416 AD corresponds to a time period during which AJ have experienced major demographic changes. At that time, AJs were speculated to have absorbed Slavic people, developed Slavic Yiddish, and intensified the migration to Europe (Das et al., 2016).
Growth of Eastern European Jewry	A small group of German Jews migrated to Eastern Europe and reproduced via a so-called “demographic miracle” (Ben-Sasson, 1976; Atzmon et al., 2010; Ostrer, 2012), which resulted in an unnatural growth rate (1.7–2% annually) (van Straten and Snel, 2006; van Straten, 2007) over half a millennium acting only on Jews residing in Eastern Europe. This explanation is unsupported by the data.	During the half millennium (740–1,250 CE), Khazar and Iranian lands harbored the largest Eurasian Jewish centers. Ashkenazic, Khazar, and Iranian Jews then sent offshoots into the Slavic lands (Baron, 1957; Sand, 2009).	Most of the Ashkenazic Jews were predicted to Northeastern Turkey and the remaining individuals clustered along a gradient going from Turkey to Eastern European lands (Das et al., 2016). This is in agreement with the recorded conversions of populations living along the southern shores of the Black Sea to Judaism (Baron, 1937). A German origin of AJs is unsupported by the data (Figure 1A).

*The genetic evidence produced by Das et al. (2016) is shown in the last column*.

## The historical meaning of ashkenaz

“Ashkenaz” is one of the most disputed Biblical placenames. It appears in the Hebrew Bible as the name of one of Noah's descendants (Genesis 10:3) and as a reference to the kingdom of Ashkenaz, prophesied to be called together with Ararat and Minnai to wage war against Babylon (Jeremiah 51:27). In addition to tracing AJs to the ancient Iranian lands of Ashkenaz and uncovering the villages whose names may derive from “Ashkenaz,” the partial Iranian origin of AJs, inferred by Das et al. (2016), was further supported by the genetic similarity of AJs to Sephardic Mountain Jews and Iranian Jews as well as their similarity to Near Eastern populations and simulated “native” Turkish and Caucasus populations.

There are good grounds, therefore, for inferring that Jews who considered themselves Ashkenazic adopted this name and spoke of their lands as Ashkenaz, since they perceived themselves as of Iranian origin. That we find varied evidence of the knowledge of Iranian language among Moroccan and Andalusian Jews and Karaites prior to the Eleventh century is a compelling point of reference to assess the shared Iranian origins of Sephardic and Ashkenazic Jews (Wexler, 1996). Moreover, Iranian-speaking Jews in the Caucasus (the so-called Juhuris) and Turkic-speaking Jews in the Crimea prior to World War II called themselves “Ashkenazim” (Weinreich, 2008).

The Rhineland hypothesis cannot explain why a name that denotes “Scythians” and was associated with the Near East became associated with German lands in the Eleventh to Thirteenth centuries (Wexler, 1993). Aptroot (2016) suggested that Jewish immigrants in Europe transferred Biblical names onto the regions in which they settled. This is unconvincing. Biblical names were used as place names only when they had similar sounds. Not only Germany and Ashkenaz do not share similar sounds, but Germany was already named “Germana,” or “Germamja” in the Iranian (“Babylonian”) Talmud (completed in the Fifth century A.D.) and, not surprisingly, was associated with Noah's grandson Gomer (Talmud, Yoma 10a). Name adoption also occurred when the exact place names were in doubt as in the case of Sefarad (Spain). This is not the case here, as Aptroot too notes, since “Ashkenaz” had a known and clear geographical affiliation (Table 1). Finally, Germany was known to French scholars like the RaDaK (1160–1235) as “Almania” (Sp. Alemania, Fr. Allemagne), after the Almani tribes, a term that was also adopted by Arab scholars. Had the French scholar Rashi (1040?-1105), interpreted aškenaz as “Germany,” it would have been known to the RaDaK who used Rashi's symbols. Therefore, Wexler's proposal that Rashi used aškenaz in the meaning of “Slavic” and that the term aškenaz assumed the solitary meaning “German lands” only after the Eleventh century in Western Europe as a result of the rise of Yiddish, is more reasonable (Wexler, 2011). This is also supported by Das et al.'s major findings of the only known primeval villages whose names derive from the word “Ashkenaz” located in the ancient lands of Ashkenaz. Our inference is therefore supported by historical, linguistic, and genetic evidence, which has more weight as a simple origin that can be easily explained than a more complex scenario that involves multiple translocations.

## The genetic structure of ashkenazic jews

AJs were localized to modern-day Turkey and found to be genetically closest to Turkic, southern Caucasian, and Iranian populations, suggesting a common origin in Iranian “Ashkenaz” lands (Das et al., 2016). These findings were more compatible with an Irano-Turko-Slavic origin for AJs and a Slavic origin for Yiddish than with the Rhineland hypothesis, which lacks historical, genetic, and linguistic support (Table 1) (van Straten, 2004; Elhaik, 2013). The findings have also highlighted the strong social-cultural and genetic bonds of Ashkenazic and Iranian Judaism and their shared Iranian origins (Das et al., 2016).

**Figure 1:**
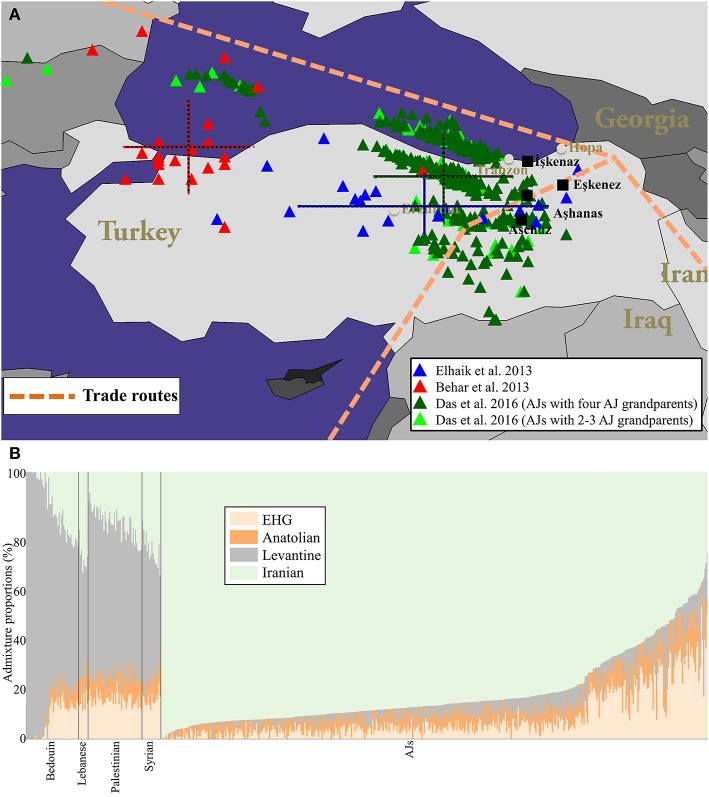
The localization of AJs and their ancient admixture proportions compared to neighboring populations. **(A)** Geographical predictions of individuals analyzed in three separate studies employing different tools: Elhaik (2013, Figure 4) (blue), Behar et al. (2013, Figure 2B) (red), and Das et al. (2016, Figure 4) (dark green for AJs who have four AJ grandparents and light green for the rest) are shown. Color matching mean and standard deviation (bars) of the longitude and latitude are shown for each cohort. Since we were unsuccessful in obtaining the data points of Behar et al. (2013, Figure 2B) from the corresponding author, we procured 78% of the data points from their figure. Due to the low quality of their figure we were unable to reliably extract the remaining data points. **(B)** Supervised ADMIXTURE results. For brevity, subpopulations were collapsed. The *x* axis represents individuals. Each individual is represented by a vertical stacked column of color-coded admixture proportions that reflect genetic contributions from ancient Hunter-Gatherer, Anatolian, Levantine, and Iranian individuals.

Thus far, all analyses aimed to geo-localize AJs (Behar et al., 2013, Figure 2B; Elhaik, 2013, Figure 4; Das et al., 2016, Figure 4) identified Turkey as the predominant origin of AJs, although they used different approaches and datasets, in support of the Irano-Turko-Slavic hypothesis (Figure 1A, Table 1). The existence of both major Southern European and Near Eastern ancestries in AJ genomes are also strong indictors of the Irano-Turko-Slavic hypothesis provided the Greco-Roman history of the region southern to the Black Sea (Baron, 1937; Kraemer, 2010). Recently, Xue et al. (2017) applied GLOBETROTTER to a dataset of 2,540 AJs genotyped over 252,358 SNPs. The inferred ancestry profile for AJs was 5% Western Europe, 10% Eastern Europe, 30% Levant, and 55% Southern Europe (a Near East ancestry was not considered by the authors). Elhaik (2013) portrayed a similar profile for European Jews, consisting of 25–30% Middle East and large Near Eastern–Caucasus (32–38%) and West European (30%) ancestries. Remarkably, Xue et al. (2017) also inferred an “admixture time” of 960–1,416 AD (≈24–40 generations ago), which corresponds to the time AJs experienced major geographical shifts as the Judaized Khazar kingdom diminished and their trading networks collapsed forcing them to relocate to Europe (Das et al., 2016). The lower boundary of that date corresponds to the time Slavic Yiddish originated, to the best of our knowledge.

The non-Levantine origin of AJs is further supported by an ancient DNA analysis of six Natufians and a Levantine Neolithic (Lazaridis et al., 2016), some of the most likely Judaean progenitors (Finkelstein and Silberman, 2002; Frendo, 2004). In a principle component analysis (PCA), the ancient Levantines clustered predominantly with modern-day Palestinians and Bedouins and marginally overlapped with Arabian Jews, whereas AJs clustered away from Levantine individuals and adjacent to Neolithic Anatolians and Late Neolithic and Bronze Age Europeans. To evaluate these findings, we inferred the ancient ancestries of AJs using the admixture analysis described in Marshall et al. (2016). Briefly, we analyzed 18,757 autosomal SNPs genotyped in 46 Palestinians, 45 Bedouins, 16 Syrians, and eight Lebanese (Li et al., 2008) alongside 467 AJs [367 AJs previously analyzed and 100 individuals with AJ mother) (Das et al., 2016) that overlapped with both the GenoChip (Elhaik et al., 2013) and ancient DNA data (Lazaridis et al., 2016). We then carried out a supervised ADMIXTURE analysis (Alexander and Lange, 2011) using three East European Hunter Gatherers from Russia (EHGs) alongside six Epipaleolithic Levantines, 24 Neolithic Anatolians, and six Neolithic Iranians as reference populations (Table S0). Remarkably, AJs exhibit a dominant Iranian ($\tilde{88\%}$) and residual Levantine ($\tilde{3\%}$) ancestries, as opposed to Bedouins ($\tilde{14\%}$ and $\tilde{68\%}$, respectively) and Palestinians ($\tilde{18\%}$ and $\tilde{58\%}$, respectively). Only two AJs exhibit Levantine ancestries typical to Levantine populations (Figure 1B). Repeating the analysis with *qpAdm* (AdmixTools, version 4.1) (Patterson et al., 2012), we found that AJs admixture could be modeled using either three- (Neolithic Anatolians [46%], Neolithic Iranians [32%], and EHGs [22%]) or two-way (Neolithic Iranians [71%] and EHGs [29%]) migration waves (Supplementary Text). These findings should be reevaluated when Medieval DNA would become available. Overall, the combined results are in a strong agreement with the predictions of the Irano-Turko-Slavic hypothesis (Table 1) and rule out an ancient Levantine origin for AJs, which is predominant among modern-day Levantine populations (e.g., Bedouins and Palestinians). This is not surprising since Jews differed in cultural practices and norms (Sand, 2011) and tended to adopt local customs (Falk, 2006). Very little Palestinian Jewish culture survived outside of Palestine (Sand, 2009). For example, the folklore and folkways of the Jews in northern Europe is distinctly pre-Christian German (Patai, 1983) and Slavic in origin, which disappeared among the latter (Wexler, 1993, 2012).

## The linguistic debate concerning formation of yiddish

The hypothesis that Yiddish has a German origin ignores the mechanics of relexification, the linguistic process which produced Yiddish and other “Old Jewish” languages (i.e., those created by the Ninth to Tenth century). Understanding how relexification operates is essential to understanding the evolution of languages. This argument has a similar context to that of the evolution of powered flight. Rejecting the theory of evolution may lead one to conclude that birds and bats are close relatives. By disregarding the literature on relexification and Jewish history in the early Middle Ages, authors (e.g., Aptroot, 2016; Flegontov et al., 2016) reach conclusions that have weak historical support. The advantage of a geo-localization analysis is that it allows us to infer the geographical origin of the speakers of Yiddish, where they resided and with whom they intermingled, independently of historical controversies, which provides a data driven view on the question of geographical origins. This allows an objective review of potential linguistic influences on Yiddish (Table 1), which exposes the dangers in adopting a “linguistic creationism” view in linguistics.

The historical evidence in favor of an Irano-Turko-Slavic origin for Yiddish is paramount (e.g., Wexler, 1993, 2010). Jews played a major role on the Silk Roads in the Ninth to Eleventh century. In the mid-Ninth century, in roughly the same years, Jewish merchants in both Mainz and at Xi'an received special trading privileges from the Holy Roman Empire and the Tang dynasty court (Robert, 2014). These roads linked Xi'an to Mainz and Andalusia, and further to sub-Saharan Africa and across to the Arabian Peninsula and India-Pakistan. The Silk Roads provided the motivation for Jewish settlement in Afro-Eurasia in the Ninth to Eleventh centuries since the Jews played a dominant role on these routes as a neutral trading guild with no political agendas (Gil, 1974; Cansdale, 1996, 1998). Hence, the Jewish traders had contact with a wealth of languages in the areas that they traversed (Hadj-Sadok, 1949; Khordadhbeh, 1889; Hansen, 2012; Wexler TBD), which they brought back to their communities nested in major trading hubs (Rabinowitz, 1945, 1948; Das et al., 2016). The central Eurasian Silk Roads were controlled by Iranian polities, which provided opportunities for Iranian-speaking Jews, who constituted the overwhelming bulk of the world's Jews from the time of Christ to the Eleventh century (Baron, 1952). It should not come as a surprise to find that Yiddish (and other Old Jewish languages) contains components and rules from a large variety of languages, all of them spoken on the Silk Roads (Khordadhbeh, 1889; Wexler, 2011, 2012, 2017).

In addition to language contacts, the Silk Roads also provided the motivation for widespread conversion to Judaism by populations eager to participate in the extremely lucrative trade, which had become a Jewish quasi-monopoly along the trade routes (Rabinowitz, 1945, 1948; Baron, 1957). These conversions are discussed in Jewish literature between the Sixth and Eleventh centuries, both in Europe and Iraq (Sand, 2009; Kraemer, 2010). Yiddish and other Old Jewish languages were all created by the peripatetic merchants as secret languages that would isolate them from their customers and non-Jewish trading partners (Hadj-Sadok, 1949; Gil, 1974; Khordadhbeh, 1889; Cansdale, 1998; Robert, 2014). The study of Yiddish genesis, thereby, necessitates the study of all the Old Jewish languages of this time period.

There is also a quantifiable amount of Iranian and Turkic elements in Yiddish. The Babylonian Talmud, completed by the Sixth century A.D., is rich in Iranian linguistic, legalistic, and religious influences. From the Talmud, a large Iranian vocabulary has entered Hebrew and Judeo-Aramaic, and from there spread to Yiddish. This corpus has been known since the 1930s and is common knowledge to Talmud scholars (Telegdi, 1933). In the Khazar Empire, the Eurasian Jews, plying the Silk Roads, became speakers of Slavic—an important language because of the trading activities of the Rus' (pre-Ukrainians) with whom the Jews were undoubtedly allied on the routes linking Baghdad and Bavaria. This is evident by the existence of newly invented Hebroidism, inspired by Slavic patterns of discourse in Yiddish (Wexler, 2010).

We advocate for implementing a more evolutionary understanding in linguistics. That includes giving more attention to the linguistic process that alter languages (e.g., relexification) and acquiring more competence in other languages and histories. When studying the origin of Ashkenazic Jews and Yiddish, such knowledge should include the history of the Silk Roads and Irano-Turkish languages.

## Inference of geographical origins

Deciphering the origin of human populations is not a new challenge for geneticists, yet only in the past decade high-throughput genetic data were harnessed to answer these questions. Here, we briefly discuss the differences between the available tools based on identity by distance. Existing PCA or PCA-like approaches (e.g., Novembre et al., 2008; Yang et al., 2012) can localize Europeans to countries (understood as the last place where major admixture event took place or the place where the four ancestors of “unmixed” individuals came from) with less than 50% accuracy (Yang et al., 2012). The limitations of PCA (discussed in Novembre and Stephens, 2008) appear to be inherent in the framework where continental populations plotted along the two primary PCs cluster in the vertices of a triangle-like shape and the remaining populations cluster along or within the edges (e.g., Elhaik et al., 2013). There is therefore reason to question the applicability of ambitious PCA-based methods (Yang et al., 2012, 2014) aiming to infer multiple ancestral locations outside of Europe. Overall, accurate localization of worldwide individuals remains a significant challenge (Elhaik et al., 2014).

The GPS framework assumes that humans are mixed and that their genetic variation (admixture) can be modeled by the proportion of genotypes assigned to any number of fixed regional *putative ancestral populations* (Elhaik et al., 2014). GPS employs a supervised ADMIXTURE analysis where the admixture components are fixed, which allows evaluating both the test individuals and *reference populations* against the same *putative ancestral populations*. GPS infers the geographical coordinates of an individual by matching their admixture proportions with those of *reference populations*. *Reference populations* are populations known to reside in a certain geographical region for a substantial period of time in a time frame of hundreds to a thousand years and can be predicted to their geographical locations while absent from the *reference population panel* (Das et al., 2016). The final geographic location of a test individual is determined by converting the genetic distance of the individual to *m reference populations* into geographic distances (Elhaik et al., 2014). Intuitively, the *reference populations* can be thought of as “pulling” the individual in their direction with a strength proportional to their genetic similarity until a consensus is reached (Figure S1). Interpreting the results, particularly when the predicted location differs from the contemporary location of the studied population, demands cautious.

Population structure is affected by biological and demographic processes like genetic drift, which can act rapidly on small, relatively isolated populations, as opposed to large non-isolated populations, and migration, which occurs more frequently (Jobling et al., 2013). Understanding the geography-admixture relationships necessitates knowing how relative isolation and migration history affected the allele frequencies of populations. Unfortunately, oftentimes we lack information about both processes. GPS addresses this problem by analyzing the relative proportions of admixture in a global network of *reference populations* that provide us with different “snapshots” of historical admixture events. These global admixture events occurred at different times through different biological and demographic processes, and their long-lasting effect is related to our ability to associate an individual with their matching admixture event.

In relatively isolated populations the admixture event is likely old, and GPS would localize a test individual with their parental population more accurately. By contrast, if the admixture event was recent and the population did not maintain relative isolation, GPS prediction would be erroneous (Figure S2). This is the case of Caribbean populations, whose admixture proportions still reflect the massive Nineteenth and Twentieth centuries' mixture events involving Native Americans, West Europeans, and Africans (Elhaik et al., 2014). While the original level of isolation remains unknown, these two scenarios can be distinguished by comparing the admixture proportions of the test individual and adjacent populations. If this similarity is high, we can conclude that we have inferred the likely location of the admixture event that shaped the admixture proportion of the test individual. If the opposite is true, the individual is either mixed and thereby violates the assumptions of the GPS model or the parental populations do not exist either in GPS's reference panel or in reality. Most of the time (83%) GPS predicted unmixed individuals to their true locations with most of the remaining individuals predicted to neighboring countries (Elhaik et al., 2014).

To understand how migration modifies the admixture proportions of the migratory and host populations, we can consider two simple cases of point or massive migration followed by assimilation and a third case of migration followed by isolation. Point migration events have little effect on the admixture proportions of the host population, particularly when it absorbs a paucity of migrants, in which case the migrants' admixture proportions would resemble those of the host population within a few generations and their resting place would represent that of the host population. Massive demographic movements, such as large-scale invasion or migration that affect a large part of the population are rare and create temporal shifts in the admixture proportions of the host population. The host population would temporarily appear as a two-way mixed population, reflecting the components of the host and invading populations (e.g., European and Native American, in the case of Puerto Ricans) until the admixture proportions would homogenize population-wise. If this process is completed, the admixture signature of this region may be altered and the geographical placement of the host population would represent again the last place where the admixture event took place for both the host and invading populations. GPS would, thereby, predict the host population's location for both populations. Populations that migrate from A to B and maintain genetic isolation would be predicted to point A in the leave-one-out population analysis. While human migrations are not uncommon, maintaining a perfect genetic isolation over a long period of time is very difficult (e.g., Veeramah et al., 2011; Behar et al., 2012; Elhaik, 2016; Hellenthal et al., 2016), and GPS predictions for the vast majority of worldwide populations indicate that these cases are indeed exceptional (Elhaik et al., 2014). Despite of its advantages, GPS has several limitations. First, it yields the most accurate predictions for unmixed individuals. Second, using migratory or highly mixed populations (both are detectable through the leave-one-out population analysis) as *reference populations* may bias the predictions. Further developments are necessary to overcome these limitations and make GPS applicable to mixed population groups (e.g., African Americans).

## Conclusion

The meaning of the term “Ashkenaz” and the geographical origins of AJs and Yiddish are some of the longest standing questions in history, genetics, and linguistics. In our previous work we have identified “ancient Ashkenaz,” a region in northeastern Turkey that harbors four primeval villages whose names resemble Ashkenaz. Here, we elaborate on the meaning of this term and argue that it acquired its modern meaning only after a critical mass of Ashkenazic Jews arrived in Germany. We show that all bio-localization analyses have localized AJs to Turkey and that the non-Levantine origins of AJs are supported by ancient genome analyses. Overall, these findings are compatible with the hypothesis of an Irano-Turko-Slavic origin for AJs and a Slavic origin for Yiddish and contradict the predictions of Rhineland hypothesis that lacks historical, genetic, and linguistic support (Table 1).

## Author contributions

EE conceived the paper. MP processed the ancient DNA data. RD and EE carried out the analyses. EE co-wrote it with PW and RD. All authors approved the paper.

### Conflict of interest statement

EE is a consultant for DNA Diagnostic Centre. The other authors declare that the research was conducted in the absence of any commercial or financial relationships that could be construed as a potential conflict of interest. The reviewer PF declared a past co-authorship with one of the authors to the handling Editor, who ensured that the process nevertheless met the standards of a fair and objective review.
