# Supplementary material for: The Origins of Ashkenaz, Ashkenazic Jews, and Yiddish
Source: Front Genet. 2017 Jun 21;8:87. doi: 10.3389/fgene.2017.00087 (PMC5478715; doi:10.3389/fgene.2017.00087)
Supplement: Supplementary file 1 [file DataSheet1.DOCX]

# Supplementary materials

**Table of Contents**

| Figure S1 – Illustration of GPS localization model for unmixed and mixed individuals | 2 |
| --- | --- |
| Figure S2 – An illustration of GPS results | 3 |
| Supplementary text – Admixture history of Ashkenazic Jews | 4-17 |

## Figure S1

**
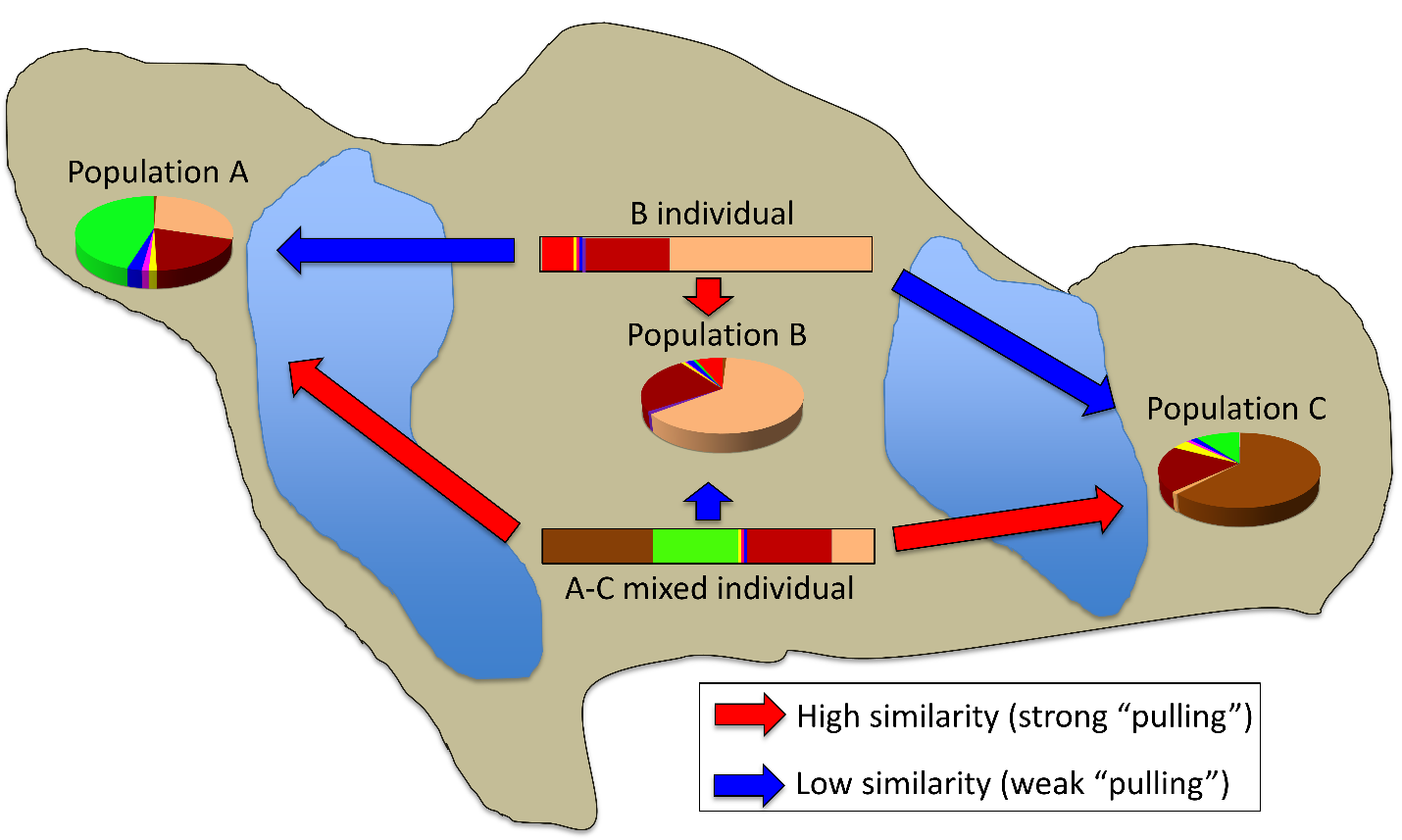
**

**Illustration of GPS localization model for unmixed and mixed individuals**. In determining the location of unmixed individual B, the individual’s admixture proportions are compared to those of three reference populations (A, B, and C). The genetic distances between individual B and populations A and C are high, thereby their “pull” is weak and their effect on the final location of this individual is minor, compared to that of the true parental population B. A-C mixed individual is predicted incorrectly to the region of population B, which happened, by chance, to reside between populations A and C, both of which are “pulling” the individual in equal strengths. Evidently, B is not A-C’s parental population since their admixture proportions are very different.

## Figure S2


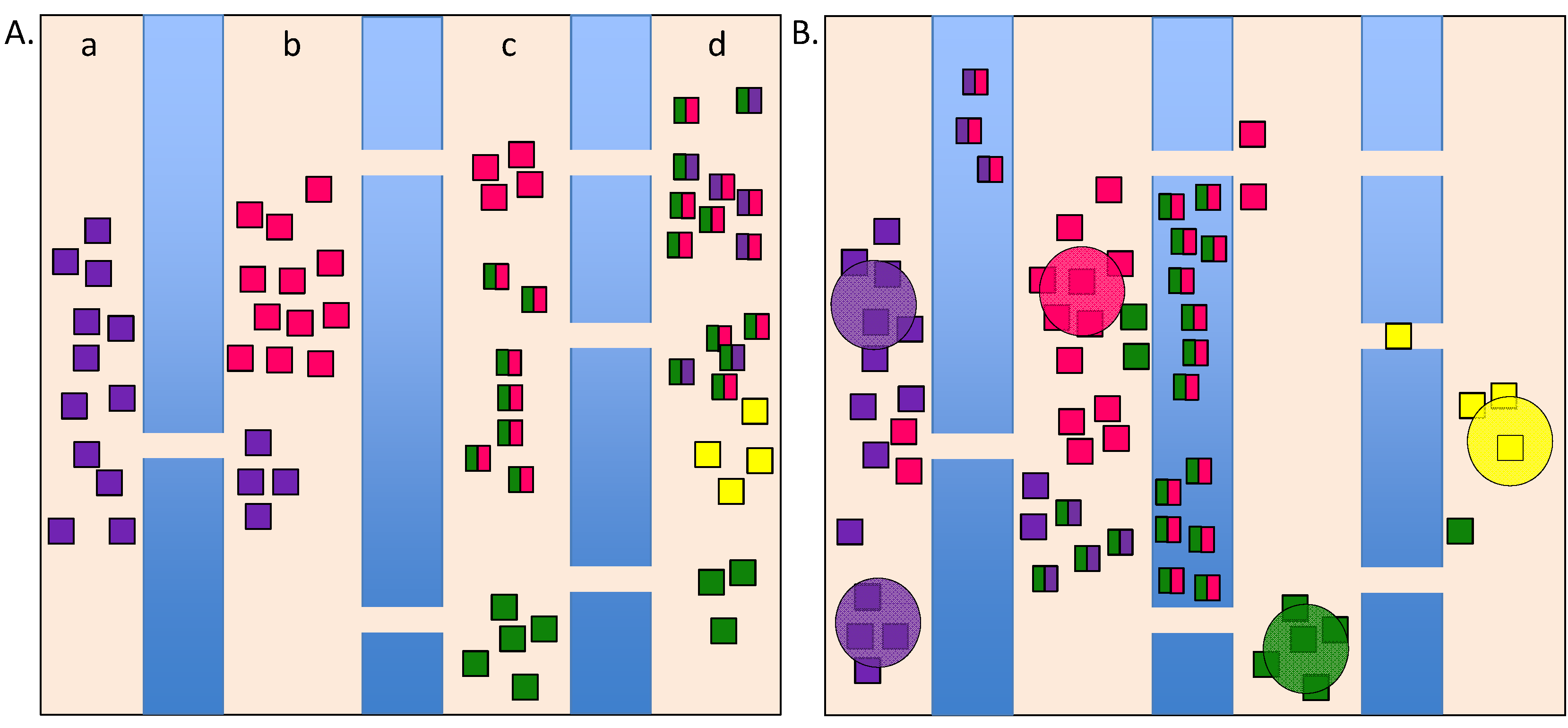


**An illustration of GPS results**. A hypothetical world consists of four regions (a-d) that vary in the degree of isolation due to natural barriers. Descendants of four unmixed populations are shown by single-color squares alongside two-ways admixed individuals shown by color-matched squares. The modern-day residency of individuals is shown in A. GPS predictions (B) are made using a panel of four reference populations (circles) positioned in the ancestral locations of the unmixed populations that gravitate genetically similar individuals towards them. GPS predicts most of the unmixed individuals to the ancestral location of their population with some inaccuracies due to the shared history of neighboring populations. The mixed individuals are predicted incorrectly to the region between their parental populations.

## **Supplementary text – Admixture history of Ashkenazic Jews**

In calculating the admixture proportions of Ashkenazic Jews we explored four-, three-, and two-way migration models involving ancient and modern-day populations. Each model was compared to the same outgroup populations and to a reduced set to test its robustness (Tables S1-23). The models most robust models that are consistent with the data are shown in Table S24. Overall we evaluated 11 migration models.

Our outgroup populations (O15) comprised of diverse non-Middle Eastern present-day populations from Africa (Mbuti, Mandenka, and San), East Asia (Han, Japanese, and Yizu), South Asia (Sindhi and Pathan), Oceania (Papuan and Melanesian), America (Karitiana, Surui, and Pima), and West Europe (Orcadian and Basque).

The migration models we tested considered AJs to be an admixture of East European Hunter Gatherers from Russia (EHGs), Neolithic Anatolians, Epipaleolithic Levantines, and Neolithic Iranians.

# Evaluating the genetic relationships between the reference populations

Prior to testing the migration models, we evaluated whether AJs, EHG, Neolithic Anatolians, Epipaleolithic Levantines, and Neolithic Iranians (Table S0) are distinguishable using the O15 outgroup populations. For all models qpWave tests have been performed on all population pairs (e.g., AJ+partner1, AJ+partner2, and partner1+partner2). The qpWave outputs (Table S1) confirmed that all populations were distinguishable given the set of outgroups (O15) (*P*-value < 0.05).

**Table S0**: Samples included in the analysis

| **ID** | **Population** | **Reference** |
| --- | --- | --- |
| I0124 | EHG | (Lazaridis et al. 2016) |
| I0211 | EHG | (Lazaridis et al. 2016) |
| I0061 | EHG | (Lazaridis et al. 2016) |
| I1100 | Anatolian | (Lazaridis et al. 2016) |
| I1102 | Anatolian | (Lazaridis et al. 2016) |
| I1099 | Anatolian | (Lazaridis et al. 2016) |
| I1103 | Anatolian | (Lazaridis et al. 2016) |
| I1101 | Anatolian | (Lazaridis et al. 2016) |
| I1097 | Anatolian | (Lazaridis et al. 2016) |
| I0744 | Anatolian | (Lazaridis et al. 2016) |
| I1579 | Anatolian | (Lazaridis et al. 2016) |
| I1581 | Anatolian | (Lazaridis et al. 2016) |
| I1096 | Anatolian | (Lazaridis et al. 2016) |
| I1580 | Anatolian | (Lazaridis et al. 2016) |
| I1098 | Anatolian | (Lazaridis et al. 2016) |
| I1585 | Anatolian | (Lazaridis et al. 2016) |
| I0708 | Anatolian | (Lazaridis et al. 2016) |
| I0745 | Anatolian | (Lazaridis et al. 2016) |
| I0746 | Anatolian | (Lazaridis et al. 2016) |
| I1583 | Anatolian | (Lazaridis et al. 2016) |
| I0707 | Anatolian | (Lazaridis et al. 2016) |
| I0709 | Anatolian | (Lazaridis et al. 2016) |
| I0736 | Anatolian | (Lazaridis et al. 2016) |
| I1290 | Iranian | (Lazaridis et al. 2016) |
| I1069 | Levantine | (Lazaridis et al. 2016) |
| I1687 | Levantine | (Lazaridis et al. 2016) |
| I1690 | Levantine | (Lazaridis et al. 2016) |
| I1685 | Levantine | (Lazaridis et al. 2016) |
| I1072 | Levantine | (Lazaridis et al. 2016) |
| I1671 | Iranian | (Lazaridis et al. 2016) |
| I0726 | Anatolian | (Lazaridis et al. 2016) |
| I0861 | Levantine | (Lazaridis et al. 2016) |
| I1944 | Iranian | (Lazaridis et al. 2016) |
| I1945 | Iranian | (Lazaridis et al. 2016) |
| I1949 | Iranian | (Lazaridis et al. 2016) |
| I1951 | Iranian | (Lazaridis et al. 2016) |
| I0723 | Anatolian | (Lazaridis et al. 2016) |
| I0724 | Anatolian | (Lazaridis et al. 2016) |
| I0727 | Anatolian | (Lazaridis et al. 2016) |
| NG33478799 | AJ | (Das et al. 2016) |
| NG3386F744 | AJ | (Das et al. 2016) |
| NG34SKC79D | AJ | (Das et al. 2016) |
| NG357Y4V7M | AJ | (Das et al. 2016) |
| NG35C39Q37 | AJ | (Das et al. 2016) |
| NG35FC3Y3V | AJ | (Das et al. 2016) |
| NG374A58G9 | AJ | (Das et al. 2016) |
| NG3793M336 | AJ | (Das et al. 2016) |
| NG37V4S87P | AJ | (Das et al. 2016) |
| NG38454345 | AJ | (Das et al. 2016) |
| NG3864783J | AJ | (Das et al. 2016) |
| NG3888DK84 | AJ | (Das et al. 2016) |
| NG38G9YV5G | AJ | (Das et al. 2016) |
| NG38NRULV4 | AJ | (Das et al. 2016) |
| NG38PV83JH | AJ | (Das et al. 2016) |
| NG39HPKP8E | AJ | (Das et al. 2016) |
| NG39HV6N5P | AJ | (Das et al. 2016) |
| NG3B7VJK8U | AJ | (Das et al. 2016) |
| NG3B85HY47 | AJ | (Das et al. 2016) |
| NG3E58LD3X | AJ | (Das et al. 2016) |
| NG3EL6T9U9 | AJ | (Das et al. 2016) |
| NG3FSSY568 | AJ | (Das et al. 2016) |
| NG3FUV89L3 | AJ | (Das et al. 2016) |
| NG3HQ39DM6 | AJ | (Das et al. 2016) |
| NG3J978C64 | AJ | (Das et al. 2016) |
| NG3L4C8Q49 | AJ | (Das et al. 2016) |
| NG3LQU4644 | AJ | (Das et al. 2016) |
| NG3P39B9LG | AJ | (Das et al. 2016) |
| NG3Q95T8QG | AJ | (Das et al. 2016) |
| NG3SFBT876 | AJ | (Das et al. 2016) |
| NG3T4G984F | AJ | (Das et al. 2016) |
| NG3T97MG99 | AJ | (Das et al. 2016) |
| NG3TYHQ84P | AJ | (Das et al. 2016) |
| NG3VJUF5H7 | AJ | (Das et al. 2016) |
| NG3YVC6M96 | AJ | (Das et al. 2016) |
| NG43BNJTVD | AJ | (Das et al. 2016) |
| NG43U3C785 | AJ | (Das et al. 2016) |
| NG44654AR3 | AJ | (Das et al. 2016) |
| NG44KNV4B4 | AJ | (Das et al. 2016) |
| NG4533U7E5 | AJ | (Das et al. 2016) |
| NG45HK8B9E | AJ | (Das et al. 2016) |
| NG464Q3SND | AJ | (Das et al. 2016) |
| NG4659NV3F | AJ | (Das et al. 2016) |
| NG47KR4465 | AJ | (Das et al. 2016) |
| NG483K4CY6 | AJ | (Das et al. 2016) |
| NG48794E94 | AJ | (Das et al. 2016) |
| NG48936AAU | AJ | (Das et al. 2016) |
| NG48DVGK6X | AJ | (Das et al. 2016) |
| NG48MX4859 | AJ | (Das et al. 2016) |
| NG498V438J | AJ | (Das et al. 2016) |
| NG4AT4N568 | AJ | (Das et al. 2016) |
| NG4C87L363 | AJ | (Das et al. 2016) |
| NG4CA38976 | AJ | (Das et al. 2016) |
| NG4DY4RUDY | AJ | (Das et al. 2016) |
| NG4HCRQNGQ | AJ | (Das et al. 2016) |
| NG4HY3SLEF | AJ | (Das et al. 2016) |
| NG4J89GL8G | AJ | (Das et al. 2016) |
| NG4KN6Q3H6 | AJ | (Das et al. 2016) |
| NG4N3Q4B6D | AJ | (Das et al. 2016) |
| NG4PJ34QFA | AJ | (Das et al. 2016) |
| NG4PMH678E | AJ | (Das et al. 2016) |
| NG4Q7L6DPH | AJ | (Das et al. 2016) |
| NG4TJN8PSM | AJ | (Das et al. 2016) |
| NG537985Y8 | AJ | (Das et al. 2016) |
| NG545ESRP7 | AJ | (Das et al. 2016) |
| NG547B9787 | AJ | (Das et al. 2016) |
| NG54834U84 | AJ | (Das et al. 2016) |
| NG548F89AP | AJ | (Das et al. 2016) |
| NG54Y44TP7 | AJ | (Das et al. 2016) |
| NG5588L4BB | AJ | (Das et al. 2016) |
| NG55JD4H65 | AJ | (Das et al. 2016) |
| NG55LN63D3 | AJ | (Das et al. 2016) |
| NG55P4M5E6 | AJ | (Das et al. 2016) |
| NG569H8458 | AJ | (Das et al. 2016) |
| NG575S659S | AJ | (Das et al. 2016) |
| NG576JVC6G | AJ | (Das et al. 2016) |
| NG57R66N79 | AJ | (Das et al. 2016) |
| NG57RJ59DU | AJ | (Das et al. 2016) |
| NG58H8L7S6 | AJ | (Das et al. 2016) |
| NG59P8695C | AJ | (Das et al. 2016) |
| NG59PH3FX8 | AJ | (Das et al. 2016) |
| NG5BME73SR | AJ | (Das et al. 2016) |
| NG5C7LM7U4 | AJ | (Das et al. 2016) |
| NG5D35Q5R4 | AJ | (Das et al. 2016) |
| NG5D55F557 | AJ | (Das et al. 2016) |
| NG5D8679KK | AJ | (Das et al. 2016) |
| NG5DL69HXC | AJ | (Das et al. 2016) |
| NG5DU3J47B | AJ | (Das et al. 2016) |
| NG5E757NQ4 | AJ | (Das et al. 2016) |
| NG5F668985 | AJ | (Das et al. 2016) |
| NG5F96H645 | AJ | (Das et al. 2016) |
| NG5H3QSY85 | AJ | (Das et al. 2016) |
| NG5HH4K63R | AJ | (Das et al. 2016) |
| NG5L4A6MU4 | AJ | (Das et al. 2016) |
| NG5L68L7YH | AJ | (Das et al. 2016) |
| NG5L8J7EDU | AJ | (Das et al. 2016) |
| NG5M3G843D | AJ | (Das et al. 2016) |
| NG5QMB3LUJ | AJ | (Das et al. 2016) |
| NG5S6S63TC | AJ | (Das et al. 2016) |
| NG5SNHYV44 | AJ | (Das et al. 2016) |
| NG5X7698S5 | AJ | (Das et al. 2016) |
| NG5Y7KJXDG | AJ | (Das et al. 2016) |
| NG5Y8755VE | AJ | (Das et al. 2016) |
| NG63334AF9 | AJ | (Das et al. 2016) |
| NG63ARMN6F | AJ | (Das et al. 2016) |
| NG63J46SBU | AJ | (Das et al. 2016) |
| NG63VKB4X8 | AJ | (Das et al. 2016) |
| NG6435B738 | AJ | (Das et al. 2016) |
| NG643A498E | AJ | (Das et al. 2016) |
| NG659A474J | AJ | (Das et al. 2016) |
| NG659DHJHB | AJ | (Das et al. 2016) |
| NG6697GFAT | AJ | (Das et al. 2016) |
| NG66UKUQR3 | AJ | (Das et al. 2016) |
| NG673K33A5 | AJ | (Das et al. 2016) |
| NG68B47P8N | AJ | (Das et al. 2016) |
| NG68MJ7969 | AJ | (Das et al. 2016) |
| NG695RLVM9 | AJ | (Das et al. 2016) |
| NG6963ADR3 | AJ | (Das et al. 2016) |
| NG6967D3U7 | AJ | (Das et al. 2016) |
| NG69F58PNH | AJ | (Das et al. 2016) |
| NG69SRK43N | AJ | (Das et al. 2016) |
| NG6BQ4TX9U | AJ | (Das et al. 2016) |
| NG6CFVQR78 | AJ | (Das et al. 2016) |
| NG6CQY6E35 | AJ | (Das et al. 2016) |
| NG6ECT9DX7 | AJ | (Das et al. 2016) |
| NG6EM799B3 | AJ | (Das et al. 2016) |
| NG6G6TDMEH | AJ | (Das et al. 2016) |
| NG6J5Y7N9H | AJ | (Das et al. 2016) |
| NG6J8RKTT8 | AJ | (Das et al. 2016) |
| NG6K398TF4 | AJ | (Das et al. 2016) |
| NG6K3X9C4L | AJ | (Das et al. 2016) |
| NG6KRHM8GF | AJ | (Das et al. 2016) |
| NG6KU49QGE | AJ | (Das et al. 2016) |
| NG6L5884KB | AJ | (Das et al. 2016) |
| NG6NS9HK58 | AJ | (Das et al. 2016) |
| NG6P78QY8H | AJ | (Das et al. 2016) |
| NG6PAJ34KC | AJ | (Das et al. 2016) |
| NG6QS44UDT | AJ | (Das et al. 2016) |
| NG6RBAKK5C | AJ | (Das et al. 2016) |
| NG6TA58Y8L | AJ | (Das et al. 2016) |
| NG733947G5 | AJ | (Das et al. 2016) |
| NG74D578AL | AJ | (Das et al. 2016) |
| NG74D9M96P | AJ | (Das et al. 2016) |
| NG75JK3L5N | AJ | (Das et al. 2016) |
| NG75L66ANM | AJ | (Das et al. 2016) |
| NG76XBA5A5 | AJ | (Das et al. 2016) |
| NG7757AG4G | AJ | (Das et al. 2016) |
| NG77677D9H | AJ | (Das et al. 2016) |
| NG776TXA65 | AJ | (Das et al. 2016) |
| NG77GLUT9D | AJ | (Das et al. 2016) |
| NG794HPCKL | AJ | (Das et al. 2016) |
| NG794N93B5 | AJ | (Das et al. 2016) |
| NG7967X7SG | AJ | (Das et al. 2016) |
| NG797X473B | AJ | (Das et al. 2016) |
| NG79M498PY | AJ | (Das et al. 2016) |
| NG7C64LYC9 | AJ | (Das et al. 2016) |
| NG7GPPREY9 | AJ | (Das et al. 2016) |
| NG7KEDRD67 | AJ | (Das et al. 2016) |
| NG7KP6B6XP | AJ | (Das et al. 2016) |
| NG7N3UFQ6M | AJ | (Das et al. 2016) |
| NG7NRS559A | AJ | (Das et al. 2016) |
| NG7Q5BELRU | AJ | (Das et al. 2016) |
| NG7QPJA4YY | AJ | (Das et al. 2016) |
| NG7R353RUJ | AJ | (Das et al. 2016) |
| NG7SY5S7FE | AJ | (Das et al. 2016) |
| NG7U3A66TU | AJ | (Das et al. 2016) |
| NG83Q5R939 | AJ | (Das et al. 2016) |
| NG8436866V | AJ | (Das et al. 2016) |
| NG853XDS3A | AJ | (Das et al. 2016) |
| NG85459L3B | AJ | (Das et al. 2016) |
| NG8577NL7Q | AJ | (Das et al. 2016) |
| NG859C5U67 | AJ | (Das et al. 2016) |
| NG85B5B7P6 | AJ | (Das et al. 2016) |
| NG85FQ89FB | AJ | (Das et al. 2016) |
| NG85MNJ53F | AJ | (Das et al. 2016) |
| NG868QXX67 | AJ | (Das et al. 2016) |
| NG87KF74JD | AJ | (Das et al. 2016) |
| NG87S8UC5X | AJ | (Das et al. 2016) |
| NG887434KC | AJ | (Das et al. 2016) |
| NG895564V6 | AJ | (Das et al. 2016) |
| NG895MX4P8 | AJ | (Das et al. 2016) |
| NG897353G4 | AJ | (Das et al. 2016) |
| NG89849QT7 | AJ | (Das et al. 2016) |
| NG898XTV59 | AJ | (Das et al. 2016) |
| NG89DX55F9 | AJ | (Das et al. 2016) |
| NG89L958KL | AJ | (Das et al. 2016) |
| NG8BUMBJBK | AJ | (Das et al. 2016) |
| NG8F335P7B | AJ | (Das et al. 2016) |
| NG8G65E4QD | AJ | (Das et al. 2016) |
| NG8GY385RX | AJ | (Das et al. 2016) |
| NG8J6DPBD4 | AJ | (Das et al. 2016) |
| NG8MA6D48V | AJ | (Das et al. 2016) |
| NG8N64RMH7 | AJ | (Das et al. 2016) |
| NG8PMN5NK6 | AJ | (Das et al. 2016) |
| NG8QD9ARQ7 | AJ | (Das et al. 2016) |
| NG8RA7JB8J | AJ | (Das et al. 2016) |
| NG8STK3977 | AJ | (Das et al. 2016) |
| NG8T444DN6 | AJ | (Das et al. 2016) |
| NG8U739KNM | AJ | (Das et al. 2016) |
| NG8YY655QV | AJ | (Das et al. 2016) |
| NG93B558M7 | AJ | (Das et al. 2016) |
| NG9448MLU3 | AJ | (Das et al. 2016) |
| NG95BCSMFJ | AJ | (Das et al. 2016) |
| NG97H9XE8Y | AJ | (Das et al. 2016) |
| NG97Y583RQ | AJ | (Das et al. 2016) |
| NG9897E76Y | AJ | (Das et al. 2016) |
| NG98ER73ES | AJ | (Das et al. 2016) |
| NG98H7DY94 | AJ | (Das et al. 2016) |
| NG98MN9E7S | AJ | (Das et al. 2016) |
| NG994LV455 | AJ | (Das et al. 2016) |
| NG998JY6MV | AJ | (Das et al. 2016) |
| NG9AEA7YK5 | AJ | (Das et al. 2016) |
| NG9BA3PC4V | AJ | (Das et al. 2016) |
| NG9CJQ5CSD | AJ | (Das et al. 2016) |
| NG9E4F36H6 | AJ | (Das et al. 2016) |
| NG9E5DEA49 | AJ | (Das et al. 2016) |
| NG9F5F3V7Y | AJ | (Das et al. 2016) |
| NG9J4E6EEN | AJ | (Das et al. 2016) |
| NG9LGV886Y | AJ | (Das et al. 2016) |
| NG9LL3JTQS | AJ | (Das et al. 2016) |
| NG9MP6Q66M | AJ | (Das et al. 2016) |
| NG9N55V74Y | AJ | (Das et al. 2016) |
| NG9NL3VBKD | AJ | (Das et al. 2016) |
| NG9PB66S99 | AJ | (Das et al. 2016) |
| NG9R5TYKQL | AJ | (Das et al. 2016) |
| NG9T9CQ99X | AJ | (Das et al. 2016) |
| NG9TFU93Y3 | AJ | (Das et al. 2016) |
| NG9TQ3NSK6 | AJ | (Das et al. 2016) |
| NG9UBRKBGS | AJ | (Das et al. 2016) |
| NG9YF8DVY9 | AJ | (Das et al. 2016) |
| NGA4DJ75S6 | AJ | (Das et al. 2016) |
| NGA4VC3SJ6 | AJ | (Das et al. 2016) |
| NGA64GY5UD | AJ | (Das et al. 2016) |
| NGA6H59JHK | AJ | (Das et al. 2016) |
| NGA8DHM968 | AJ | (Das et al. 2016) |
| NGA99A6749 | AJ | (Das et al. 2016) |
| NGAPKQ5B3M | AJ | (Das et al. 2016) |
| NGB63L3M79 | AJ | (Das et al. 2016) |
| NGB65D94D4 | AJ | (Das et al. 2016) |
| NGB6969BAE | AJ | (Das et al. 2016) |
| NGB6S3A75T | AJ | (Das et al. 2016) |
| NGB75EBKX9 | AJ | (Das et al. 2016) |
| NGB87V4QBG | AJ | (Das et al. 2016) |
| NGB8CDJFDV | AJ | (Das et al. 2016) |
| NGB97UT494 | AJ | (Das et al. 2016) |
| NGBBX578Y9 | AJ | (Das et al. 2016) |
| NGBFX4598T | AJ | (Das et al. 2016) |
| NGBKU944E7 | AJ | (Das et al. 2016) |
| NGBL6TLFEE | AJ | (Das et al. 2016) |
| NGC3958A4K | AJ | (Das et al. 2016) |
| NGC4JJJNCB | AJ | (Das et al. 2016) |
| NGC55CP98P | AJ | (Das et al. 2016) |
| NGC7H863EJ | AJ | (Das et al. 2016) |
| NGC7VF63Y7 | AJ | (Das et al. 2016) |
| NGC8TBMAGN | AJ | (Das et al. 2016) |
| NGCC8588YP | AJ | (Das et al. 2016) |
| NGCK684K5E | AJ | (Das et al. 2016) |
| NGD35AP33Y | AJ | (Das et al. 2016) |
| NGD3G3QC35 | AJ | (Das et al. 2016) |
| NGD4CMMFGS | AJ | (Das et al. 2016) |
| NGD4FU573G | AJ | (Das et al. 2016) |
| NGD4KDYBTT | AJ | (Das et al. 2016) |
| NGD6H9RKS8 | AJ | (Das et al. 2016) |
| NGD6Y47VN7 | AJ | (Das et al. 2016) |
| NGD83Y5GE6 | AJ | (Das et al. 2016) |
| NGD9659LJL | AJ | (Das et al. 2016) |
| NGDAFC35LT | AJ | (Das et al. 2016) |
| NGDKFJU6AD | AJ | (Das et al. 2016) |
| NGDU763FV5 | AJ | (Das et al. 2016) |
| NGE595L4H9 | AJ | (Das et al. 2016) |
| NGE74KS4MD | AJ | (Das et al. 2016) |
| NGE7U677M3 | AJ | (Das et al. 2016) |
| NGED33S5CA | AJ | (Das et al. 2016) |
| NGED584547 | AJ | (Das et al. 2016) |
| NGEN75S3JS | AJ | (Das et al. 2016) |
| NGEN96G4SD | AJ | (Das et al. 2016) |
| NGEP7N9HC4 | AJ | (Das et al. 2016) |
| NGF37A4B3U | AJ | (Das et al. 2016) |
| NGF4J54384 | AJ | (Das et al. 2016) |
| NGF635S5BL | AJ | (Das et al. 2016) |
| NGF6E5U95C | AJ | (Das et al. 2016) |
| NGF79M3PD8 | AJ | (Das et al. 2016) |
| NGF7D4785H | AJ | (Das et al. 2016) |
| NGF84SMA66 | AJ | (Das et al. 2016) |
| NGF8H6G47M | AJ | (Das et al. 2016) |
| NGF9S3ALF3 | AJ | (Das et al. 2016) |
| NGFAU54Y58 | AJ | (Das et al. 2016) |
| NGFGS57385 | AJ | (Das et al. 2016) |
| NGFJHYHVL7 | AJ | (Das et al. 2016) |
| NGFKL9A99X | AJ | (Das et al. 2016) |
| NGFLX7JAFR | AJ | (Das et al. 2016) |
| NGFS669388 | AJ | (Das et al. 2016) |
| NGG349XY8V | AJ | (Das et al. 2016) |
| NGG35BRYGT | AJ | (Das et al. 2016) |
| NGG5H89XG5 | AJ | (Das et al. 2016) |
| NGG8U3964D | AJ | (Das et al. 2016) |
| NGGC473FLD | AJ | (Das et al. 2016) |
| NGGCD6GU98 | AJ | (Das et al. 2016) |
| NGGEC66FV7 | AJ | (Das et al. 2016) |
| NGGFDXE6U6 | AJ | (Das et al. 2016) |
| NGGL67U5P7 | AJ | (Das et al. 2016) |
| NGGLYS7N6A | AJ | (Das et al. 2016) |
| NGGN855Q3Y | AJ | (Das et al. 2016) |
| NGH47JR77B | AJ | (Das et al. 2016) |
| NGH4TPE6XB | AJ | (Das et al. 2016) |
| NGH795CUNX | AJ | (Das et al. 2016) |
| NGH84TPCSQ | AJ | (Das et al. 2016) |
| NGH9LYUNCQ | AJ | (Das et al. 2016) |
| NGHBX4D56S | AJ | (Das et al. 2016) |
| NGHFD6A3X8 | AJ | (Das et al. 2016) |
| NGHJ635VFJ | AJ | (Das et al. 2016) |
| NGHS36B5N3 | AJ | (Das et al. 2016) |
| NGHUM5655X | AJ | (Das et al. 2016) |
| NGJ36G5JE3 | AJ | (Das et al. 2016) |
| NGJ49B96GL | AJ | (Das et al. 2016) |
| NGJ49XY39L | AJ | (Das et al. 2016) |
| NGJ57AVH9T | AJ | (Das et al. 2016) |
| NGJ5B3XDFX | AJ | (Das et al. 2016) |
| NGJ7R3ALAF | AJ | (Das et al. 2016) |
| NGJ84DD3V3 | AJ | (Das et al. 2016) |
| NGJ8FU44QJ | AJ | (Das et al. 2016) |
| NGJ96K8GFQ | AJ | (Das et al. 2016) |
| NGJ9D9YEJ9 | AJ | (Das et al. 2016) |
| NGJM49X8X4 | AJ | (Das et al. 2016) |
| NGJR446X6E | AJ | (Das et al. 2016) |
| NGK3449CH6 | AJ | (Das et al. 2016) |
| NGK37D6T35 | AJ | (Das et al. 2016) |
| NGK39P4MML | AJ | (Das et al. 2016) |
| NGK3K9TF3N | AJ | (Das et al. 2016) |
| NGK5643T7J | AJ | (Das et al. 2016) |
| NGK5889E3V | AJ | (Das et al. 2016) |
| NGK6QV5S5T | AJ | (Das et al. 2016) |
| NGK74S5E46 | AJ | (Das et al. 2016) |
| NGK83585XU | AJ | (Das et al. 2016) |
| NGK97FN36K | AJ | (Das et al. 2016) |
| NGK9MF9GR6 | AJ | (Das et al. 2016) |
| NGKC4TTSBD | AJ | (Das et al. 2016) |
| NGKG54M84T | AJ | (Das et al. 2016) |
| NGKQ3K7566 | AJ | (Das et al. 2016) |
| NGKS3L6KCC | AJ | (Das et al. 2016) |
| NGL3SH573H | AJ | (Das et al. 2016) |
| NGL665GGC5 | AJ | (Das et al. 2016) |
| NGL7344H89 | AJ | (Das et al. 2016) |
| NGL73YUF33 | AJ | (Das et al. 2016) |
| NGLDD73V86 | AJ | (Das et al. 2016) |
| NGLK5R3K98 | AJ | (Das et al. 2016) |
| NGLQH3AE44 | AJ | (Das et al. 2016) |
| NGLS863VB6 | AJ | (Das et al. 2016) |
| NGLUUL9956 | AJ | (Das et al. 2016) |
| NGLV97SU9F | AJ | (Das et al. 2016) |
| NGLYEVB856 | AJ | (Das et al. 2016) |
| NGLYQ7X4UA | AJ | (Das et al. 2016) |
| NGM35X68BF | AJ | (Das et al. 2016) |
| NGM5GF7389 | AJ | (Das et al. 2016) |
| NGM74R4P6T | AJ | (Das et al. 2016) |
| NGM75KP3U6 | AJ | (Das et al. 2016) |
| NGM7735G55 | AJ | (Das et al. 2016) |
| NGM7F49JYG | AJ | (Das et al. 2016) |
| NGM89VNMVD | AJ | (Das et al. 2016) |
| NGMD6PF4CS | AJ | (Das et al. 2016) |
| NGMJ9L3FVB | AJ | (Das et al. 2016) |
| NGMK975635 | AJ | (Das et al. 2016) |
| NGMU46CMTK | AJ | (Das et al. 2016) |
| NGMU6T4385 | AJ | (Das et al. 2016) |
| NGN3G4F57E | AJ | (Das et al. 2016) |
| NGN6A5Y4T3 | AJ | (Das et al. 2016) |
| NGN7GUB8V5 | AJ | (Das et al. 2016) |
| NGN873E6L9 | AJ | (Das et al. 2016) |
| NGNBLU39XC | AJ | (Das et al. 2016) |
| NGNR3TC4GK | AJ | (Das et al. 2016) |
| NGNX368PEX | AJ | (Das et al. 2016) |
| NGNX557873 | AJ | (Das et al. 2016) |
| NGP4B959T3 | AJ | (Das et al. 2016) |
| NGP4M9749G | AJ | (Das et al. 2016) |
| NGP5QQY4ED | AJ | (Das et al. 2016) |
| NGP6657JQU | AJ | (Das et al. 2016) |
| NGP73AFT6U | AJ | (Das et al. 2016) |
| NGP9648BS9 | AJ | (Das et al. 2016) |
| NGPVB4E368 | AJ | (Das et al. 2016) |
| NGQ39X4U93 | AJ | (Das et al. 2016) |
| NGQ447FT45 | AJ | (Das et al. 2016) |
| NGQ475U5RR | AJ | (Das et al. 2016) |
| NGQ488545S | AJ | (Das et al. 2016) |
| NGQ53T4U56 | AJ | (Das et al. 2016) |
| NGQ66MF5JX | AJ | (Das et al. 2016) |
| NGQ8939U34 | AJ | (Das et al. 2016) |
| NGQ8SHRF93 | AJ | (Das et al. 2016) |
| NGQ9974R6U | AJ | (Das et al. 2016) |
| NGQ9AH5SXY | AJ | (Das et al. 2016) |
| NGQ9V4LC4P | AJ | (Das et al. 2016) |
| NGQB8PLL5A | AJ | (Das et al. 2016) |
| NGQEQEFTQS | AJ | (Das et al. 2016) |
| NGQFG55U7V | AJ | (Das et al. 2016) |
| NGQFK4FV6T | AJ | (Das et al. 2016) |
| NGQG78XX3P | AJ | (Das et al. 2016) |
| NGQP78879U | AJ | (Das et al. 2016) |
| NGR58U45D9 | AJ | (Das et al. 2016) |
| NGR6MJQF6X | AJ | (Das et al. 2016) |
| NGR6T79NEV | AJ | (Das et al. 2016) |
| NGR99PPN7M | AJ | (Das et al. 2016) |
| NGRFYDSHM5 | AJ | (Das et al. 2016) |
| NGRG3Y3463 | AJ | (Das et al. 2016) |
| NGRG9573LC | AJ | (Das et al. 2016) |
| NGRHHJARRL | AJ | (Das et al. 2016) |
| NGRS9U7UP9 | AJ | (Das et al. 2016) |
| NGRSFU5Q67 | AJ | (Das et al. 2016) |
| NGRT5J98BM | AJ | (Das et al. 2016) |
| NGSCERTR46 | AJ | (Das et al. 2016) |
| NGSEVJ3566 | AJ | (Das et al. 2016) |
| NGSL398T6J | AJ | (Das et al. 2016) |
| NGSN3AE583 | AJ | (Das et al. 2016) |
| NGSSKHB3R7 | AJ | (Das et al. 2016) |
| NGSTJEQ56Y | AJ | (Das et al. 2016) |
| NGSUK6TP8M | AJ | (Das et al. 2016) |
| NGT44FVFAR | AJ | (Das et al. 2016) |
| NGT73UU5RG | AJ | (Das et al. 2016) |
| NGT7P878VP | AJ | (Das et al. 2016) |
| NGT88MCGX4 | AJ | (Das et al. 2016) |
| NGT99VS57L | AJ | (Das et al. 2016) |
| NGT9FE5R74 | AJ | (Das et al. 2016) |
| NGTBG49KT6 | AJ | (Das et al. 2016) |
| NGTE7HLY48 | AJ | (Das et al. 2016) |
| NGTF9686X6 | AJ | (Das et al. 2016) |
| NGTJU43T5D | AJ | (Das et al. 2016) |
| NGTMP3T6H9 | AJ | (Das et al. 2016) |
| NGTR75T75H | AJ | (Das et al. 2016) |
| NGTS49A7UP | AJ | (Das et al. 2016) |
| NGTV6CV43G | AJ | (Das et al. 2016) |
| NGTV79FJB8 | AJ | (Das et al. 2016) |
| NGTX35X4MH | AJ | (Das et al. 2016) |
| NGU3LN49JE | AJ | (Das et al. 2016) |
| NGU6X534RD | AJ | (Das et al. 2016) |
| NGU7399QJ8 | AJ | (Das et al. 2016) |
| NGUC8HBFA6 | AJ | (Das et al. 2016) |
| NGUDBURP8Y | AJ | (Das et al. 2016) |
| NGUEVV39H7 | AJ | (Das et al. 2016) |
| NGUG6LX377 | AJ | (Das et al. 2016) |
| NGUJG65FVK | AJ | (Das et al. 2016) |
| NGUKE6BGHV | AJ | (Das et al. 2016) |
| NGUM5756Q7 | AJ | (Das et al. 2016) |
| NGUM5YF3JN | AJ | (Das et al. 2016) |
| NGUU47N4TT | AJ | (Das et al. 2016) |
| NGV5KQ6MTL | AJ | (Das et al. 2016) |
| NGV7375UU9 | AJ | (Das et al. 2016) |
| NGV959HEG8 | AJ | (Das et al. 2016) |
| NGV9K3KL7L | AJ | (Das et al. 2016) |
| NGVGL3K98Y | AJ | (Das et al. 2016) |
| NGVP77JU9G | AJ | (Das et al. 2016) |
| NGVS8RF434 | AJ | (Das et al. 2016) |
| NGX3783K5Q | AJ | (Das et al. 2016) |
| NGX3P7N5P8 | AJ | (Das et al. 2016) |
| NGX47P6N33 | AJ | (Das et al. 2016) |
| NGX95U79RP | AJ | (Das et al. 2016) |
| NGX974555C | AJ | (Das et al. 2016) |
| NGXCV69833 | AJ | (Das et al. 2016) |
| NGXN66UM79 | AJ | (Das et al. 2016) |
| NGXV598E87 | AJ | (Das et al. 2016) |
| NGY443A9CG | AJ | (Das et al. 2016) |
| NGY5J4MJDU | AJ | (Das et al. 2016) |
| NGY73D9MX9 | AJ | (Das et al. 2016) |
| NGY93R96V6 | AJ | (Das et al. 2016) |
| NGY9TSBGV6 | AJ | (Das et al. 2016) |
| NGYA63CP53 | AJ | (Das et al. 2016) |
| NGYAJD48LN | AJ | (Das et al. 2016) |
| NGYAXE8AKH | AJ | (Das et al. 2016) |
| NGYD77FA35 | AJ | (Das et al. 2016) |
| NGYX9XDBB6 | AJ | (Das et al. 2016) |
| HGDP00607 | Bedouin | (Li et al. 2008) |
| HGDP00608 | Bedouin | (Li et al. 2008) |
| HGDP00609 | Bedouin | (Li et al. 2008) |
| HGDP00610 | Bedouin | (Li et al. 2008) |
| HGDP00611 | Bedouin | (Li et al. 2008) |
| HGDP00612 | Bedouin | (Li et al. 2008) |
| HGDP00613 | Bedouin | (Li et al. 2008) |
| HGDP00614 | Bedouin | (Li et al. 2008) |
| HGDP00615 | Bedouin | (Li et al. 2008) |
| HGDP00616 | Bedouin | (Li et al. 2008) |
| HGDP00618 | Bedouin | (Li et al. 2008) |
| HGDP00619 | Bedouin | (Li et al. 2008) |
| HGDP00620 | Bedouin | (Li et al. 2008) |
| HGDP00622 | Bedouin | (Li et al. 2008) |
| HGDP00623 | Bedouin | (Li et al. 2008) |
| HGDP00624 | Bedouin | (Li et al. 2008) |
| HGDP00625 | Bedouin | (Li et al. 2008) |
| HGDP00626 | Bedouin | (Li et al. 2008) |
| HGDP00627 | Bedouin | (Li et al. 2008) |
| HGDP00628 | Bedouin | (Li et al. 2008) |
| HGDP00629 | Bedouin | (Li et al. 2008) |
| HGDP00630 | Bedouin | (Li et al. 2008) |
| HGDP00631 | Bedouin | (Li et al. 2008) |
| HGDP00632 | Bedouin | (Li et al. 2008) |
| HGDP00634 | Bedouin | (Li et al. 2008) |
| HGDP00635 | Bedouin | (Li et al. 2008) |
| HGDP00636 | Bedouin | (Li et al. 2008) |
| HGDP00637 | Bedouin | (Li et al. 2008) |
| HGDP00638 | Bedouin | (Li et al. 2008) |
| HGDP00639 | Bedouin | (Li et al. 2008) |
| HGDP00640 | Bedouin | (Li et al. 2008) |
| HGDP00641 | Bedouin | (Li et al. 2008) |
| HGDP00642 | Bedouin | (Li et al. 2008) |
| HGDP00643 | Bedouin | (Li et al. 2008) |
| HGDP00644 | Bedouin | (Li et al. 2008) |
| HGDP00645 | Bedouin | (Li et al. 2008) |
| HGDP00646 | Bedouin | (Li et al. 2008) |
| HGDP00647 | Bedouin | (Li et al. 2008) |
| HGDP00648 | Bedouin | (Li et al. 2008) |
| HGDP00649 | Bedouin | (Li et al. 2008) |
| HGDP00650 | Bedouin | (Li et al. 2008) |
| HGDP00651 | Bedouin | (Li et al. 2008) |
| HGDP00653 | Bedouin | (Li et al. 2008) |
| HGDP00654 | Bedouin | (Li et al. 2008) |
| HGDP00701 | Bedouin | (Li et al. 2008) |
| Lebanon1 | Lebanese | (Li et al. 2008) |
| Lebanon2 | Lebanese | (Li et al. 2008) |
| Lebanon3 | Lebanese | (Li et al. 2008) |
| Lebanon4 | Lebanese | (Li et al. 2008) |
| Lebanon5 | Lebanese | (Li et al. 2008) |
| Lebanon6 | Lebanese | (Li et al. 2008) |
| Lebanon7 | Lebanese | (Li et al. 2008) |
| Lebanon8 | Lebanese | (Li et al. 2008) |
| HGDP00675 | Palestinian | (Li et al. 2008) |
| HGDP00676 | Palestinian | (Li et al. 2008) |
| HGDP00677 | Palestinian | (Li et al. 2008) |
| HGDP00678 | Palestinian | (Li et al. 2008) |
| HGDP00679 | Palestinian | (Li et al. 2008) |
| HGDP00680 | Palestinian | (Li et al. 2008) |
| HGDP00682 | Palestinian | (Li et al. 2008) |
| HGDP00683 | Palestinian | (Li et al. 2008) |
| HGDP00684 | Palestinian | (Li et al. 2008) |
| HGDP00685 | Palestinian | (Li et al. 2008) |
| HGDP00686 | Palestinian | (Li et al. 2008) |
| HGDP00687 | Palestinian | (Li et al. 2008) |
| HGDP00688 | Palestinian | (Li et al. 2008) |
| HGDP00689 | Palestinian | (Li et al. 2008) |
| HGDP00690 | Palestinian | (Li et al. 2008) |
| HGDP00691 | Palestinian | (Li et al. 2008) |
| HGDP00692 | Palestinian | (Li et al. 2008) |
| HGDP00693 | Palestinian | (Li et al. 2008) |
| HGDP00694 | Palestinian | (Li et al. 2008) |
| HGDP00696 | Palestinian | (Li et al. 2008) |
| HGDP00697 | Palestinian | (Li et al. 2008) |
| HGDP00698 | Palestinian | (Li et al. 2008) |
| HGDP00699 | Palestinian | (Li et al. 2008) |
| HGDP00700 | Palestinian | (Li et al. 2008) |
| HGDP00722 | Palestinian | (Li et al. 2008) |
| HGDP00723 | Palestinian | (Li et al. 2008) |
| HGDP00724 | Palestinian | (Li et al. 2008) |
| HGDP00725 | Palestinian | (Li et al. 2008) |
| HGDP00726 | Palestinian | (Li et al. 2008) |
| HGDP00727 | Palestinian | (Li et al. 2008) |
| HGDP00729 | Palestinian | (Li et al. 2008) |
| HGDP00730 | Palestinian | (Li et al. 2008) |
| HGDP00731 | Palestinian | (Li et al. 2008) |
| HGDP00732 | Palestinian | (Li et al. 2008) |
| HGDP00733 | Palestinian | (Li et al. 2008) |
| HGDP00734 | Palestinian | (Li et al. 2008) |
| HGDP00735 | Palestinian | (Li et al. 2008) |
| HGDP00736 | Palestinian | (Li et al. 2008) |
| HGDP00737 | Palestinian | (Li et al. 2008) |
| HGDP00738 | Palestinian | (Li et al. 2008) |
| HGDP00739 | Palestinian | (Li et al. 2008) |
| HGDP00740 | Palestinian | (Li et al. 2008) |
| HGDP00741 | Palestinian | (Li et al. 2008) |
| HGDP00744 | Palestinian | (Li et al. 2008) |
| HGDP00745 | Palestinian | (Li et al. 2008) |
| HGDP00746 | Palestinian | (Li et al. 2008) |
| syria1 | Syrians | (Behar et al. 2010) |
| syria2 | Syrians | (Behar et al. 2010) |
| syria3 | Syrians | (Behar et al. 2010) |
| syria4 | Syrians | (Behar et al. 2010) |
| syria5 | Syrians | (Behar et al. 2010) |
| syria6 | Syrians | (Behar et al. 2010) |
| syria7 | Syrians | (Behar et al. 2010) |
| syria8 | Syrians | (Behar et al. 2010) |
| syria9 | Syrians | (Behar et al. 2010) |
| syria10 | Syrians | (Behar et al. 2010) |
| syria298 | Syrians | (Behar et al. 2010) |
| syria361 | Syrians | (Behar et al. 2010) |
| syria461 | Syrians | (Behar et al. 2010) |
| syria464 | Syrians | (Behar et al. 2010) |
| syria485 | Syrians | (Behar et al. 2010) |
| syria520 | Syrians | (Behar et al. 2010) |

**Table S1**: Pairwise qpWave comparison among different population pairs given O15 reference populations

| **Left population pairs** | **f4rank 0 Chi-square** | ***P*-value** |
| --- | --- | --- |
| 1. AJ - EHG | 159.107 | 1.07x10^-26^ |
| 2. AJ - Iranian | 67.131 | 6.36x10^-09^ |
| 3. AJ - Anatolian | 110.583 | 4.31x10^-17^ |
| 4. AJ - Levantine | 86.388 | 1.82x10^-12^ |
| 5. EHG - Iranian | 188.237 | 1.37x10^-32^ |
| 6. EHG - Anatolian | 210.386 | 4.12x10^-37^ |
| 7. EHG - Levantine | 170.9 | 4.51x10^-29^ |
| 8. Iranian - Anatolian | 54.641 | 9.98x10^-07^ |
| 9. Iranian - Levantine | 37.201 | 0.000687737 |
| 10. Anatolian - Levantine | 38.386 | 0.000453448 |

## Evaluating four-way models

We first considered AJs as an admixture of four populations: **AJs = EHGs + Iranians + Anatolians + Levantines** (Table S2).

**Table S2**: qpWave and qpAdm results for the four-way migration model. The admixture coefficients and std. errors are shown for: EHGs, Iranians, Anatolians, and Levantines

| **qpWave ranks** | **Chi-square** | ***P-value*** | **Admixture coefficients** | **Std. errors** |
| --- | --- | --- | --- | --- |
| f4rank 0 | 427.766 | 1.12x10^-58^ | 0.180 0.351 0.728 -0.259 | 0.05 0.10 0.25 0.21 |
| f4rank 1 | 105.268 | 5.29x10^-08^ |  |  |
| f4rank 2 | 32.116 | 0.124 |  |  |
| f4rank 3 | 8.329 | 0.684 |  |  |
| f4rank 4 | 0 | 1 |  |  |

To evaluate the robustness of the model we examined the effect of dropping each of the geographic regions on the output of qpWave and qpAdm (Table S3). Overall, regardless of which geographical region is dropped, the admixture coefficients obtained from qpAdm outputs indicate potential infeasibility of the four-way migration model.

**Table S3**: qpWave and qpAdm results after dropping each geographic region. The admixture coefficients and std. errors are shown for: EHGs, Iranians, Anatolians, and Levantines

| **Region dropped** | | **qpWave ranks** | | **Chi-square** | | ***P-value*** | | **Admixture coefficients** | | **Std. errors** |
| --- | --- | --- | --- | --- | --- | --- | --- | --- | --- | --- |
| Oceania | f4rank 0 | | 370.988 | | 1.81x10^-51^ | | 0.20 0.33 0.69 -0.23 | | 0.05 0.10 0.22 0.19 | |
|  | f4rank 1 | | 85.876 | | 1.36x10^-06^ | |  | |  | |
|  | f4rank 2 | | 26.628 | | 0.146 | |  | |  | |
|  | f4rank 3 | | 6.362 | | 0.703 | |  | |  | |
|  | f4rank 4 | | 0 | | 1 | |  | |  | |
|  |  | |  | |  | |  | |  | |
| East Asia | f4rank 0 | | 340.259 | | 2.03x10^-47^ | | 0.13 0.44 0.83 -0.4 | | 0.08 0.16 0.30 0.27 | |
|  | f4rank 1 | | 85.393 | | 3.22x10^-07^ | |  | |  | |
|  | f4rank 2 | | 27.259 | | 0.074 | |  | |  | |
|  | f4rank 3 | | 5.263 | | 0.729 | |  | |  | |
|  | f4rank 4 | | 0 | | 1 | |  | |  | |
| Africa | f4rank 0 | | 302.48 | | 2.79x10^-40^ | | 0.18 0.34 0.76 -0.27 | | 0.06 0.15 0.37 0.26 | |
|  | f4rank 1 | | 80.691 | | 1.57x10^-06^ | |  | |  | |
|  | f4rank 2 | | 26.947 | | 0.079 | |  | |  | |
|  | f4rank 3 | | 8.095 | | 0.424 | |  | |  | |
|  | f4rank 4 | | 0 | | 1 | |  | |  | |
| America | f4rank 0 | | 355.174 | | 2.87x10^-50^ | | 0.16 0.29 0.89 -0.34 | | 0.2 0.23 1.1 0.71 | |
|  | f4rank 1 | | 77.639 | | 4.29x10^-06^ | |  | |  | |
|  | f4rank 2 | | 16.419 | | 0.563 | |  | |  | |
|  | f4rank 3 | | 6.572 | | 0.583 | |  | |  | |
|  | f4rank 4 | | 0 | | 1 | |  | |  | |
| South Asia | f4rank 0 | | 406.841 | | 2.44x10^-58^ | | 0.21 0.35 0.60 -0.16 | | 0.04 0.09 0.20 0.17 | |
|  | f4rank 1 | | 95.362 | | 5.71x10^-08^ | |  | |  | |
|  | f4rank 2 | | 26.506 | | 0.15 | |  | |  | |
|  | f4rank 3 | | 4.416 | | 0.881 | |  | |  | |
|  | f4rank 4 | | 0 | | 1 | |  | |  | |
| Europe | f4rank 0 | | 376.559 | | 1.57x10^-52^ | | 0.17 0.47 0.81 -0.45 | | 0.07 0.19 0.31 0.32 | |
|  | f4rank 1 | | 78.654 | | 1.35x10^-05^ | |  | |  | |
|  | f4rank 2 | | 23.73 | | 0.254 | |  | |  | |
|  | f4rank 3 | | 4.859 | | 0.846 | |  | |  | |
|  | f4rank 4 | | 0 | | 1 | |  | |  | |

## Evaluating three-way models

We next evaluated three way models using the same O15 as outgroup populations. The first model explored was: **AJs = EHGs + Iranians + Anatolians** (Table S4).

**Table S4**: qpWave and qpAdm results for the three-way migration model. The admixture coefficients and std. errors are shown for: EHGs, Iranians, and Anatolians

| **qpWave ranks** | **Chi-square** | ***P-value*** | **Admixture coefficients** | **Std. errors** |
| --- | --- | --- | --- | --- |
| f4rank 0 | 366.647 | 2.05x10^-53^ | 0.225 0.318 0.457 | 0.031 0.098 0.108 |
| f4rank 1 | 78.856 | 3.142x10^-07^ |  |  |
| f4rank 2 | 11.875 | 0.456 |  |  |
| f4rank 3 | 0 | 1 |  |  |

As before, the robustness of the model was evaluated by dropping each of the geographic regions (Table S5). Overall, regardless of which geographical region is dropped, the model remains supported by the data. AJs can thereby be modelled as the genomic admixture of EHGs (~22%), Neolithic Iranians (~32%), and Neolithic Anatolians (~46%).

**Table S5**: qpWave and qpAdm results after dropping each geographic region. The admixture coefficients and std. errors are shown for: EHGs, Iranians, and Anatolians

| **Region dropped** | **qpWave ranks** | **Chi-square** | ***P-value*** | **Admixture coefficients** | **Std. errors** |
| --- | --- | --- | --- | --- | --- |
| Oceania | f4rank 0 | 317.298 | 1.011x10^-46^ | 0.241 0.285 0.474 | 0.033 0.089 0.097 |
|  | f4rank 1 | 64.521 | 4.675x10^-06^ |  |  |
|  | f4rank 2 | 9.143 | 0.518 |  |  |
|  | f4rank 3 | 0 | 1 |  |  |
| East Asia | f4rank 0 | 287.51 | 2.213x10^-42^ | 0.216 0.353 0.431 | 0.034 0.128 0.133 |
|  | f4rank 1 | 68.845 | 2.808x10^-07^ |  |  |
|  | f4rank 2 | 11.011 | 0.275 |  |  |
|  | f4rank 3 | 0 | 1 |  |  |
| Africa | f4rank 0 | 260.332 | 3.835x10^-37^ | 0.222 0.356 0.421 | 0.037 0.144 0.148 |
|  | f4rank 1 | 58.133 | 1.379x10^-05^ |  |  |
|  | f4rank 2 | 11.119 | 0.268 |  |  |
|  | f4rank 3 | 0 | 1 |  |  |
| America | f4rank 0 | 303.841 | 1.472x10^-45^ | 0.243 0.346 0.411 | 0.038 0.121 0.142 |
|  | f4rank 1 | 58.666 | 1.143x10^-05^ |  |  |
|  | f4rank 2 | 8.583 | 0.477 |  |  |
|  | f4rank 3 | 0 | 1 |  |  |
| South Asia | f4rank 0 | 350.304 | 3.659x10^-53^ | 0.234 0.319 0.447 | 0.030 0.081 0.092 |
|  | f4rank 1 | 70.029 | 6.545x10^-07^ |  |  |
|  | f4rank 2 | 5.905 | 0.823 |  |  |
|  | f4rank 3 | 0 | 1 |  |  |
| Europe | f4rank 0 | 337.867 | 9.977x10^-51^ | 0.235 0.362 0.403 | 0.035 0.129 0.146 |
|  | f4rank 1 | 65.46 | 3.356x10^-06^ |  |  |
|  | f4rank 2 | 10.724 | 0.379 |  |  |
|  | f4rank 3 | 0 | 1 |  |  |

The three-way model **AJs = EHGs + Iranians + Levantines** was next explored using the same O15 outgroup (Table S6).

**Table S6**: qpWave and qpAdm results for the three-way migration model. The admixture coefficients and std. errors are shown for: EHGs, Iranians, and Levantines

| **qpWave ranks** | **Chi-square** | ***P-value*** | **Admixture coefficients** | **Std. errors** |
| --- | --- | --- | --- | --- |
| f4rank 0 | 307.297 | 4.741x10^-42^ | 0.293 0.592 0.116 | 0.045 0.301 0.285 |
| f4rank 1 | 52.571 | 0.001 |  |  |
| f4rank 2 | 18.763 | 0.094 |  |  |
| f4rank 3 | 0 | 1 |  |  |

The robustness of the model was evaluated as before by dropping each of the geographic regions and examining the outputs of qpWave and qpAdm outputs (Table S7). While the model was mostly supported by the data, it was also sensitive to the drop of geographic regions (Europe and East Asia).

**Table S7**: qpWave and qpAdm results after dropping each geographic region. The admixture coefficients and std. errors are shown for: EHGs, Iranians, and Levantines

| **Region dropped** | **qpWave ranks** | **Chi-square** | ***P-value*** | **Admixture coefficients** | **Std. errors** |
| --- | --- | --- | --- | --- | --- |
| Oceania | f4rank 0 | 242.967 | 1.552x10^-32^ | 0.312 0.562 0.126 | 0.051 0.388 0.367 |
|  | f4rank 1 | 46.484 | 0.002 |  |  |
|  | f4rank 2 | 17.361 | 0.067 |  |  |
|  | f4rank 3 | 0 | 1 |  |  |
| East Asia | f4rank 0 | 222.87 | 4.813x10^-30^ | 0.242 0.942 -0.184 | 0.122 0.872 0.768 |
|  | f4rank 1 | 40.345 | 0.0045 |  |  |
|  | f4rank 2 | 16.583 | 0.055 |  |  |
|  | f4rank 3 | 0 | 1 |  |  |
| Africa | f4rank 0 | 219.82 | 1.79x10^-29^ | 0.258 0.621 0.121 | 0.052 0.228 0.213 |
|  | f4rank 1 | 45.335 | 0.0009 |  |  |
|  | f4rank 2 | 14.329 | 0.111 |  |  |
|  | f4rank 3 | 0 | 1 |  |  |
| America | f4rank 0 | 267.563 | 1.572x10^-38^ | 0.315 0.466 0.219 | 0.040 0.203 0.194 |
|  | f4rank 1 | 40.598 | 0.0042 |  |  |
|  | f4rank 2 | 10.119 | 0.341 |  |  |
|  | f4rank 3 | 0 | 1 |  |  |
| South Asia | f4rank 0 | 286.968 | 7.154x10^-41^ | 0.305 0.561 0.134 | 0.040 0.191 0.186 |
|  | f4rank 1 | 48.151 | 0.001 |  |  |
|  | f4rank 2 | 13.61 | 0.192 |  |  |
|  | f4rank 3 | 0 | 1 |  |  |
| Europe | f4rank 0 | 275.883 | 9.402x10^-39^ | 0.298 1.172 -0.470 | 0.074 1.481 1.466 |
|  | f4rank 1 | 34.139 | 0.0475 |  |  |
|  | f4rank 2 | 13.915 | 0.177 |  |  |
|  | f4rank 3 | 0 | 1 |  |  |

We next explored the three-way model: **AJs = EHGs + Anatolians + Levantines** using O15 as outgroup populations (Table S8).

**Table S8**: qpWave and qpAdm results for the three-way migration model. The admixture coefficients and std. errors are shown for: EHGs, Anatolians, and Levantines

| **qpWave ranks** | **Chi-square** | ***P-value*** | **Admixture coefficients** | **Std. errors** |
| --- | --- | --- | --- | --- |
| f4rank 0 | 331.461 | 1.206x10^-46^ | -0.081 2.395 -1.315 | 0.352 1.968 1.627 |
| f4rank 1 | 65.189 | 3.239x10^-05^ |  |  |
| f4rank 2 | 18.628 | 0.098 |  |  |
| f4rank 3 | 0 | 1 |  |  |

The results of the robustness analysis are summarized in Table S9. Overall, regardless of which geographic region is dropped, the admixture coefficients obtained from qpAdm outputs indicate potential infeasibility of this migration model.

**Table S9**: qpWave and qpAdm results after dropping each geographic region. The admixture coefficients and std. errors are shown for: EHGs, Anatolians, and Levantines

| **Region dropped** | **qpWave ranks** | **Chi-square** | ***P-value*** | **Admixture coefficients** | **Std. errors** |
| --- | --- | --- | --- | --- | --- |
| Oceania | f4rank 0 | 294.797 | 2.247x10^-42^ | -0.088 2.530 -1.442 | 0.800 4.490 3.697 |
|  | f4rank 1 | 49.589 | 0.0007 |  |  |
|  | f4rank 2 | 17.637 | 0.061 |  |  |
|  | f4rank 3 | 0 | 1 |  |  |
| East Asia | f4rank 0 | 278.889 | 1.032x10^-40^ | -0.546 4.343 -2.797 | 2.113 9.920 7.817 |
|  | f4rank 1 | 57.758 | 1.574x10^-05^ |  |  |
|  | f4rank 2 | 14.933 | 0.093 |  |  |
|  | f4rank 3 | 0 | 1 |  |  |
| Africa | f4rank 0 | 252.19 | 1.379x10^-35^ | 0.060 1.939 -0.999 | 0.191 1.314 1.142 |
|  | f4rank 1 | 50.705 | 0.0002 |  |  |
|  | f4rank 2 | 14.692 | 0.1 |  |  |
|  | f4rank 3 | 0 | 1 |  |  |
| America | f4rank 0 | 277.432 | 1.974x10^-40^ | -0.144 2.586 -1.442 | 0.362 1.815 1.465 |
|  | f4rank 1 | 47.739 | 0.0005 |  |  |
|  | f4rank 2 | 7.879 | 0.546 |  |  |
|  | f4rank 3 | 0 | 1 |  |  |
| South Asia | f4rank 0 | 321.988 | 1.241x10^-47^ | -0.031 2.187 -1.157 | 0.265 1.522 1.269 |
|  | f4rank 1 | 58.355 | 3.901x10^-05^ |  |  |
|  | f4rank 2 | 17.575 | 0.063 |  |  |
|  | f4rank 3 | 0 | 1 |  |  |
| Europe | f4rank 0 | 296.971 | 8.582x10^-43^ | -0.372 4.382 -3.010 | 1.512 9.636 8.138 |
|  | f4rank 1 | 56.059 | 8.387x10^-05^ |  |  |
|  | f4rank 2 | 13.866 | 0.179 |  |  |
|  | f4rank 3 | 0 | 1 |  |  |

The last three-way model we explored was: **AJs = Iranians + Anatolians + Levantines** using O15 as outgroup populations (Table S10).

**Table S10**: qpWave and qpAdm results for the three-way migration model. The admixture coefficients and std. errors are shown for: Iranians, Anatolians, and Levantines

| **qpWave ranks** | **Chi-square** | ***P-value*** | **Admixture coefficients** | **Std. errors** |
| --- | --- | --- | --- | --- |
| f4rank 0 | 227.425 | 2.682x10^-27^ | 0.313 1.511 -0.824 | 0.196 0.260 0.237 |
| f4rank 1 | 68.52 | 1.087x10^-05^ |  |  |
| f4rank 2 | 13.469 | 0.336 |  |  |
| f4rank 3 | 0 | 1 |  |  |

The results of the robustness analysis are summarized in Table S11. The results show that regardless which geographic region is dropped, the admixture coefficients obtained from qpAdm outputs indicate potential infeasibility of this model.

**Table S11**: qpWave and qpAdm results after dropping each geographic region. The admixture coefficients and std. errors are shown for: Iranians, Anatolians, and Levantines.

| **Region dropped** | **qpWave ranks** | **Chi-square** | ***P-value*** | **Admixture coefficients** | **Std. errors** |
| --- | --- | --- | --- | --- | --- |
| Oceania | f4rank 0 | 212.061 | 8.088x10^-27^ | 0.331 1.538 -0.869 | 0.226 0.292 0.268 |
|  | f4rank 1 | 60.45 | 1.917x10^-05^ |  |  |
|  | f4rank 2 | 12.88 | 0.23 |  |  |
|  | f4rank 3 | 0 | 1 |  |  |
| East Asia | f4rank 0 | 204.467 | 1.272x10^-26^ | 0.469 1.250 -0.718 | 0.207 0.235 0.205 |
|  | f4rank 1 | 50.798 | 0.0002 |  |  |
|  | f4rank 2 | 7.339 | 0.602 |  |  |
|  | f4rank 3 | 0 | 1 |  |  |
| Africa | f4rank 0 | 158.113 | 2.899x10^-18^ | 0.226 1.731 -0.957 | 0.271 0.479 0.396 |
|  | f4rank 1 | 50.917 | 0.0002 |  |  |
|  | f4rank 2 | 12.52 | 0.186 |  |  |
|  | f4rank 3 | 0 | 1 |  |  |
| America | f4rank 0 | 204.11 | 1.481x10^-26^ | 0.125 1.627 -0.751 | 0.165 0.242 0.191 |
|  | f4rank 1 | 54.468 | 4.929x10^-05^ |  |  |
|  | f4rank 2 | 5.864 | 0.753 |  |  |
|  | f4rank 3 | 0 | 1 |  |  |
| South Asia | f4rank 0 | 220.884 | 1.948x10^-28^ | 0.312 1.532 -0.844 | 0.201 0.273 0.250 |
|  | f4rank 1 | 67.618 | 1.559x10^-06^ |  |  |
|  | f4rank 2 | 12.745 | 0.238 |  |  |
|  | f4rank 3 | 0 | 1 |  |  |
| Europe | f4rank 0 | 195.42 | 8.413x10^-24^ | 0.573 1.565 -1.139 | 0.361 0.334 0.445 |
|  | f4rank 1 | 47.564 | 0.0012 |  |  |
|  | f4rank 2 | 6.874 | 0.737 |  |  |
|  | f4rank 3 | 0 | 1 |  |  |

## Evaluating two-way models

The first two-way model explored was: **AJs = EHGs + Iranians** using O15 as outgroup populations (Table S12).

**Table S12**: qpWave and qpAdm results for the two-way migration model. The admixture coefficients and std. errors are shown for: EHGs and Iranians.

| **qpWave ranks** | **Chi-square** | ***P-value*** | **Admixture coefficients** | **Std. errors** |
| --- | --- | --- | --- | --- |
| f4rank 0 | 233.507 | 2.66x10^-34^ | 0.292 0.708 | 0.049 0.049 |
| f4rank 1 | 20.399 | 0.0857 |  |  |
| f4rank 2 | 0 | 1 |  |  |

As before, we evaluated the robustness of the model by dropping each of the geographic regions and examining the effect of the output of qpWave and qpAdm (Table S13). We found that regardless which geographical region is dropped the model is supported by the data. AJ can thereby be modelled as the genomic admixtures of EHGs (~29%) and Neolithic Iranians (~71%).

**Table S13**: qpWave and qpAdm results after dropping each geographic region. The admixture coefficients and std. errors are shown for: EHGs and Iranians.

| **Region dropped** | **qpWave ranks** | **Chi-square** | ***P-value*** | **Admixture coefficients** | **Std. errors** |
| --- | --- | --- | --- | --- | --- |
| Oceania | f4rank 0 | 178.467 | 1.44x10^-25^ | 0.311 0.689 | 0.057 0.057 |
|  | f4rank 1 | 19.012 | 0.0601 |  |  |
|  | f4rank 2 | 0 | 1 |  |  |
| East Asia | f4rank 0 | 163.865 | 1.12x10^-23^ | 0.258 0.742 | 0.054 0.054 |
|  | f4rank 1 | 16.64 | 0.0827 |  |  |
|  | f4rank 2 | 0 | 1 |  |  |
| Africa | f4rank 0 | 171.813 | 3.35x10^-25^ | 0.267 0.733 | 0.060 0.060 |
|  | f4rank 1 | 16.446 | 0.0876 |  |  |
|  | f4rank 2 | 0 | 1 |  |  |
| America | f4rank 0 | 201.099 | 6.93x10^-31^ | 0.318 0.682 | 0.051 0.051 |
|  | f4rank 1 | 13.18 | 0.213 |  |  |
|  | f4rank 2 | 0 | 1 |  |  |
| South Asia | f4rank 0 | 215.691 | 9.32x10^-33^ | 0.310 0.690 | 0.046 0.046 |
|  | f4rank 1 | 15.505 | 0.161 |  |  |
|  | f4rank 2 | 0 | 1 |  |  |
| Europe | f4rank 0 | 226.951 | 5.82x10^-35^ | 0.300 0.700 | 0.046 0.046 |
|  | f4rank 1 | 15.244 | 0.172 |  |  |
|  | f4rank 2 | 0 | 1 |  |  |

We next explored the model: **AJs = EHGs + Anatolians** using O15 as outgroup (Table S14).

**Table S14**: qpWave and qpAdm results for the two-way migration model. The admixture coefficients and std. errors are shown for: EHGs and Anatolians

| **qpWave ranks** | **Chi-square** | ***P-value*** | **Admixture coefficients** | **Std. errors** |
| --- | --- | --- | --- | --- |
| f4rank 0 | 279.996 | 2.22x10^-43^ | 0.210 0.790 | 0.032 0.032 |
| f4rank 1 | 42.807 | 4.83x10^-05^ |  |  |
| f4rank 2 | 0 | 1 |  |  |

The results of the robustness analysis are summarized in Table S15. We found that the data do not support this two-way migration model. The f4rank 1 *P*-value<0.05, which indicates that the data do not support this two-way migration model.

**Table S15**: qpWave and qpAdm results after dropping each geographic region. The admixture coefficients and std. errors are shown for: EHGs and Anatolians

| **Region dropped** | **qpWave ranks** | **Chi-square** | ***P-value*** | **Admixture coefficients** | **Std. errors** |
| --- | --- | --- | --- | --- | --- |
| Oceania | f4rank 0 | 248.882 | 2.22x10^-43^ | 0.240 0.760 | 0.037 0.037 |
|  | f4rank 1 | 31.387 | 0.0009 |  |  |
|  | f4rank 2 | 0 | 1 |  |  |
| East Asia | f4rank 0 | 232.702 | 4.04x10^-37^ | 0.228 0.772 | 0.039 0.039 |
|  | f4rank 1 | 40.449 | 1.41x10^-05^ |  |  |
|  | f4rank 2 | 0 | 1 |  |  |
| Africa | f4rank 0 | 215.179 | 1.86x10^-33^ | 0.223 0.777 | 0.035 0.035 |
|  | f4rank 1 | 29.295 | 0.001 |  |  |
|  | f4rank 2 | 0 | 1 |  |  |
| America | f4rank 0 | 237.892 | 3.75x10^-38^ | 0.208 0.792 | 0.031 0.031 |
|  | f4rank 1 | 35.806 | 9.09x10^-05^ |  |  |
|  | f4rank 2 | 0 | 1 |  |  |
| South Asia | f4rank 0 | 270.955 | 1.12x10^-43^ | 0.218 0.782 | 0.033 0.033 |
|  | f4rank 1 | 36.755 | 0.0001 |  |  |
|  | f4rank 2 | 0 | 1 |  |  |
| Europe | f4rank 0 | 255.545 | 1.31x10^-40^ | 0.204 0.796 | 0.033 0.033 |
|  | f4rank 1 | 41.583 | 1.91x10^-05^ |  |  |
|  | f4rank 2 | 0 | 1 |  |  |

We next explored the model: **AJs = EHGs + Levantines** using O15 as outgroup populations (Table S16).

**Table S16**: qpWave and qpAdm results for the two-way migration model. The admixture coefficients and std. errors are shown for: EHGs and Levantines

| **qpWave ranks** | **Chi-square** | ***P-value*** | **Admixture coefficients** | **Std. errors** |
| --- | --- | --- | --- | --- |
| f4rank 0 | 225.883 | 7.86x10^-33^ | 0.334 0.666 | 0.043 0.043 |
| f4rank 1 | 28.581 | 0.0075 |  |  |
| f4rank 2 | 0 | 1 |  |  |

The results of the robustness analysis are summarized in Table S17. Overall, the data do not support this two-way migration model. The f4rank 1 *P*-value<0.05, which indicates that the data do not support this two-way migration model.

**Table S17**: qpWave and qpAdm results after dropping each geographic region. The admixture coefficients and std. errors are shown for: EHGs and Levantines

| **Region dropped** | **qpWave ranks** | **Chi-square** | ***P-value*** | **Admixture coefficients** | **Std. errors** |
| --- | --- | --- | --- | --- | --- |
| Oceania | f4rank 0 | 177.198 | 2.51x10^-25^ | 0.355 0.645 | 0.043 0.043 |
|  | f4rank 1 | 22.155 | 0.023 |  |  |
|  | f4rank 2 | 0 | 1 |  |  |
| East Asia | f4rank 0 | 178.243 | 1.93x10^-26^ | 0.366 0.634 | 0.045 0.045 |
|  | f4rank 1 | 23.144 | 0.01 |  |  |
|  | f4rank 2 | 0 | 1 |  |  |
| Africa | f4rank 0 | 173.269 | 1.76x10^-25^ | 0.308 0.692 | 0.056 0.056 |
|  | f4rank 1 | 27.03 | 0.002 |  |  |
|  | f4rank 2 | 0 | 1 |  |  |
| America | f4rank 0 | 195.36 | 9.18x10^-30^ | 0.340 0.660 | 0.043 0.043 |
|  | f4rank 1 | 21.254 | 0.019 |  |  |
|  | f4rank 2 | 0 | 1 |  |  |
| South Asia | f4rank 0 | 215.705 | 9.26x10^-33^ | 0.334 0.666 | 0.044 0.044 |
|  | f4rank 1 | 27.157 | 0.004 |  |  |
|  | f4rank 2 | 0 | 1 |  |  |
| Europe | f4rank 0 | 205.896 | 7.52x10^-31^ | 0.314 0.686 | 0.046 0.046 |
|  | f4rank 1 | 21.588 | 0.028 |  |  |
|  | f4rank 2 | 0 | 1 |  |  |

We next explored the model: **AJs = Iranians + Anatolians** using O15 as outgroup populations (Table S18).

**Table S18**: qpWave and qpAdm results for the two-way migration model. The admixture coefficients and std. errors are shown for: Iranians and Anatolians

| **qpWave ranks** | **Chi-square** | ***P-value*** | **Admixture coefficients** | **Std. errors** |
| --- | --- | --- | --- | --- |
| f4rank 0 | 170.41 | 2.34x10^-22^ | -2.640 3.640 | 4.254 4.254 |
| f4rank 1 | 53.505 | 7.38x10^-07^ |  |  |
| f4rank 2 | 0 | 1 |  |  |

The results of the robustness analysis are summarized in Table S19. We found that the data do not support this model. The f4rank 1 *P*-value<0.05, which indicates that the data do not support this two-way migration model.

**Table S19**: qpWave and qpAdm results after dropping each geographic region. The admixture coefficients and std. errors are shown for: Iranian and Anatolian

| **Region dropped** | **qpWave ranks** | **Chi-square** | ***P-value*** | **Admixture coefficients** | **Std. errors** |
| --- | --- | --- | --- | --- | --- |
| Oceania | f4rank 0 | 158.93 | 7.13x10^-22^ | -2.566 3.566 | 4.044 4.044 |
|  | f4rank 1 | 47.595 | 1.69x10^-06^ |  |  |
|  | f4rank 2 | 0 | 1 |  |  |
| East Asia | f4rank 0 | 142.27 | 1.36x10^-19^ | 4.254 -3.254 | 8.779 8.779 |
|  | f4rank 1 | 35.956 | 8.57x10^-05^ |  |  |
|  | f4rank 2 | 0 | 1 |  |  |
| Africa | f4rank 0 | 118.961 | 2.69x10^-15^ | -7.643 8.643 | 57.595 57.595 |
|  | f4rank 1 | 40.174 | 1.58x10^-05^ |  |  |
|  | f4rank 2 | 0 | 1 |  |  |
| America | f4rank 0 | 154.786 | 5.96x10^-22^ | -1.925 2.925 | 2.274 2.274 |
|  | f4rank 1 | 46.395 | 1.21x10^-06^ |  |  |
|  | f4rank 2 | 0 | 1 |  |  |
| South Asia | f4rank 0 | 167.068 | 2.09x10^-23^ | -2.503 3.503 | 3.917 3.917 |
|  | f4rank 1 | 53.278 | 1.59x10^-07^ |  |  |
|  | f4rank 2 | 0 | 1 |  |  |
| Europe | f4rank 0 | 155.544 | 3.07x10^-21^ | -1.634 2.634 | 1.407 1.407 |
|  | f4rank 1 | 42.366 | 1.4x10^-05^ |  |  |
|  | f4rank 2 | 0 | 1 |  |  |

We next explored the model: **AJs = Iranians+ Levantines** using O15 as outgroup populations (Table S20).

**Table S20**: qpWave and qpAdm results for the two-way migration model. The admixture coefficients and std. errors are shown for: Iranians and Levantines

| **qpWave ranks** | **Chi-square** | ***P-value*** | **Admixture coefficients** | **Std. errors** |
| --- | --- | --- | --- | --- |
| f4rank 0 | 145.071 | 9.45x10^-18^ | 4.184 -3.184 | 4.596 4.596 |
| f4rank 1 | 35.913 | 0.0006 |  |  |
| f4rank 2 | 0 | 1 |  |  |

The results of the robustness analysis are summarized in Table S21. We found that the data do not support this two-way migration model. The f4rank 1 *P*-value<0.05, which indicates that the data do not support this two-way migration model.

**Table S21**: qpWave and qpAdm results after dropping each geographic region. The admixture coefficients and std. errors are shown for: Iranians and Levantines

| **Region dropped** | **qpWave ranks** | **Chi-square** | ***P-value*** | **Admixture coefficients** | **Std. errors** |
| --- | --- | --- | --- | --- | --- |
| Oceania | f4rank 0 | 134.331 | 2.54x10^-17^ | 3.991 -2.991 | 4.117 4.117 |
|  | f4rank 1 | 31.84 | 0.0008 |  |  |
|  | f4rank 2 | 0 | 1 |  |  |
| East Asia | f4rank 0 | 118.894 | 2.77x10^-15^ | 2.372 -1.372 | 1.060 1.060 |
|  | f4rank 1 | 21.499 | 0.018 |  |  |
|  | f4rank 2 | 0 | 1 |  |  |
| Africa | f4rank 0 | 98.637 | 1.12x10^-11^ | 3.116 -2.116 | 3.598 3.598 |
|  | f4rank 1 | 30.652 | 0.0007 |  |  |
|  | f4rank 2 | 0 | 1 |  |  |
| America | f4rank 0 | 134.599 | 3.64x10^-18^ | 4.385 -3.385 | 5.598 5.598 |
|  | f4rank 1 | 35.365 | 0.0001 |  |  |
|  | f4rank 2 | 0 | 1 |  |  |
| South Asia | f4rank 0 | 141.201 | 1.4x10^-18^ | 4.473 -3.473 | 5.413 5.413 |
|  | f4rank 1 | 35.504 | 0.0002 |  |  |
|  | f4rank 2 | 0 | 1 |  |  |
| Europe | f4rank 0 | 118.304 | 1.94x10^-14^ | 12.651 -11.651 | 36.582 36.582 |
|  | f4rank 1 | 17.062 | 0.106 |  |  |
|  | f4rank 2 | 0 | 1 |  |  |

The final model we explored was: **AJs = Anatolians + Levantines** using O15 as outgroup populations (Table S22).

**Table S22**: qpWave and qpAdm results for the two-way migration model. The admixture coefficients and std. errors are shown for: Anatolians and Levantines

| **qpWave ranks** | **Chi-square** | ***P-value*** | **Admixture coefficients** | **Std. errors** |
| --- | --- | --- | --- | --- |
| f4rank 0 | 169.398 | 3.59x10^-22^ | 1.832 -0.832 | 0.239 0.239 |
| f4rank 1 | 17.88 | 0.162 |  |  |
| f4rank 2 | 0 | 1 |  |  |

The results of the robustness analysis are summarized in Table S23. We found that the data do not support this two-way migration model. Although the f4rank 1 *P*-value>0.05, the qpAdm output generated negative admixture coefficients, which indicates that the data do not support this two-way migration model.

**Table S23**: qpWave and qpAdm results after dropping each geographic region. The admixture coefficients and std. errors are shown for: Anatolians and Levantines

| **Region dropped** | **qpWave ranks** | **Chi-square** | ***P-value*** | **Admixture coefficients** | **Std. errors** |
| --- | --- | --- | --- | --- | --- |
| Oceania | f4rank 0 | 163.474 | 9.98x10^-23^ | 1.877 -0.877 | 0.270 0.270 |
|  | f4rank 1 | 16.139 | 0.136 |  |  |
|  | f4rank 2 | 0 | 1 |  |  |
| East Asia | f4rank 0 | 163.34 | 1.41x10^-23^ | 1.790 -0.790 | 0.233 0.233 |
|  | f4rank 1 | 16.664 | 0.082 |  |  |
|  | f4rank 2 | 0 | 1 |  |  |
| Africa | f4rank 0 | 128.261 | 5.39x10^-17^ | 1.966 -0.966 | 0.382 0.382 |
|  | f4rank 1 | 13.855 | 0.179 |  |  |
|  | f4rank 2 | 0 | 1 |  |  |
| America | f4rank 0 | 147.778 | 1.25x10^-20^ | 1.735 -0.735 | 0.182 0.182 |
|  | f4rank 1 | 8.426 | 0.587 |  |  |
|  | f4rank 2 | 0 | 1 |  |  |
| South Asia | f4rank 0 | 165.865 | 3.54x10^-23^ | 1.851 -0.851 | 0.251 0.251 |
|  | f4rank 1 | 16.794 | 0.114 |  |  |
|  | f4rank 2 | 0 | 1 |  |  |
| Europe | f4rank 0 | 151.03 | 2.13x10^-20^ | 1.978 -0.978 | 0.335 0.335 |
|  | f4rank 1 | 13.473 | 0.264 |  |  |
|  | f4rank 2 | 0 | 1 |  |  |

## Conclusions

Of the 11 migration models we evaluated, only two are consistent with the data (Table S24). To summarize these analyses, a Four-way migration model comprising of East European Hunter Gatherers from Russia (EHG), Neolithic Anatolians, Epipaleolithic Levantines, and Neolithic Iranians cannot be used to model the genomic admixture of AJs.

Three-way migration models comprising of Epipaleolithic Levantines alongside any two populations out of EHG, Neolithic Anatolians, or Neolithic Iranians are unfeasible. Of all three-way migration models and irrespective of which geographic region is dropped, the only model supported by the data depicted AJs as the genomic admixture of Neolithic Iranians (32%), Neolithic Anatolians (46%), and EHG (22%).

Of the two-way models, we found that AJs cannot be modelled as the genomic admixtures of Neolithic Iranians and Neolithic Anatolians, or genomic admixture of EHGs and Neolithic Anatolians. None of the two-way models comprising of Levantine populations, irrespective of which geographical region is dropped, is supported by the data. Therefore, AJs cannot be modelled as a genomic admixture of Epipaleolithic Levantines and Neolithic Iranians, Neolithic Anatolians, or EHG. The models are not supported either due to negative *P*-values or due to negative admixture coefficients both of which are indicators that the data do not support the model. By contrast, AJs could be modelled as EHG (29%) and Neolithic Iranians (71%). This model is very similar to the three-way model supported by the data, likely due to the genetic similarity between Iranians and Anatolians.

The two most supported three- (Iranian, Anatolian, and EHG) and two-way (Iranian and EHG) models were insensitive to the drop of geographic regions. We note, however, that these results may change with the inclusions of new ancient reference populations found in the region.

**Table S24**: Summarizing the most supported models for AJs

| **Migration model / table** | **Source populations** | **Reference populations** | **Chi-square** | ***P*-value** | **Admixture components** | **Standard Errors** |
| --- | --- | --- | --- | --- | --- | --- |
| Three-way | Iranians  Anatolians  EHGs | O15 | 11.875 (f4 rank 2) | 0.46 | 0.318  0.457  0.225 | 0.09  0.11  0.03 |
| Two-way | Iranians  EHGs | O15 | 0.086  (f4 rank 1) |  | 0.708  0.292 | 0.05 0.05 |

**REFERENCES**

Behar DM, et al. 2010. The genome-wide structure of the Jewish people. Nature. 466:238-242.

Das R, et al. 2016. Localizing Ashkenazic Jews to primeval villages in the ancient Iranian lands of Ashkenaz. Genome Biol. Evol. 8:1132–1149.

Lazaridis I, et al. 2016. Genomic insights into the origin of farming in the ancient Near East. Nature. 536:419-424.

Li JZ, et al. 2008. Worldwide human relationships inferred from genome-wide patterns of variation. Science. 319:1100-1104.
